# Ecology and molecular targets of hypermutation in the global microbiome

**DOI:** 10.1038/s41467-021-23402-7

**Published:** 2021-05-24

**Authors:** Simon Roux, Blair G. Paul, Sarah C. Bagby, Stephen Nayfach, Michelle A. Allen, Graeme Attwood, Ricardo Cavicchioli, Ludmila Chistoserdova, Robert J. Gruninger, Steven J. Hallam, Maria E. Hernandez, Matthias Hess, Wen-Tso Liu, Tim A. McAllister, Michelle A. O’Malley, Xuefeng Peng, Virginia I. Rich, Scott R. Saleska, Emiley A. Eloe-Fadrosh

**Affiliations:** 1 DOE Joint Genome Institute, Lawrence Berkeley National Laboratory, Berkeley, CA USA; 2 Marine Biological Laboratory, Woods Hole, MA USA; 3 Department of Biology, Case Western Reserve University, Cleveland, OH USA; 4 The University of New South Wales, Sydney, NSW Australia; 5 AgResearch Limited, Grasslands Research Centre, Palmerston North, New Zealand; 6 University of Washington, Seattle, WA USA; 7 Lethbridge Research and Development Centre, Agriculture and Agri-Food Canada, Lethbridge, Alberta Canada; 8 Department of Microbiology and Immunology, University of British Columbia, Vancouver, Canada; 9 Graduate Program in Bioinformatics, University of British Columbia, Genome Sciences Centre, Vancouver, Canada; 10 Genome Science and Technology Program, University of British Columbia, Vancouver, Canada; 11 Life Sciences Institute, University of British Columbia, Vancouver, Canada; 12 ECOSCOPE Training Program, University of British Columbia, Vancouver, Canada; 13 Instituto de Ecología A.C. Red de Manejo Biotechnológico de Recursos. Xalapa, Veracruz, México; 14 University of California Davis, Davis, CA USA; 15 University of Illinois at Urbana-Champaign, Urbana, IL USA; 16 Department of Chemical Engineering, University of California Santa Barbara, Santa Barbara, CA USA; 17 Marine Science Institute, University of California Santa Barbara, Santa Barbara, CA USA; 18 Ohio State University, Columbus, OH USA; 19 University of Arizona, Tucson, AZ USA

**Keywords:** Molecular evolution, Environmental microbiology, Phage biology

## Abstract

Changes in the sequence of an organism’s genome, i.e., mutations, are the raw material of evolution. The frequency and location of mutations can be constrained by specific molecular mechanisms, such as diversity-generating retroelements (DGRs). DGRs have been characterized from cultivated bacteria and bacteriophages, and perform error-prone reverse transcription leading to mutations being introduced in specific target genes. DGR loci were also identified in several metagenomes, but the ecological roles and evolutionary drivers of these DGRs remain poorly understood. Here, we analyze a dataset of >30,000 DGRs from public metagenomes, establish six major lineages of DGRs including three primarily encoded by phages and seemingly used to diversify host attachment proteins, and demonstrate that DGRs are broadly active and responsible for >10% of all amino acid changes in some organisms. Overall, these results highlight the constraints under which DGRs evolve, and elucidate several distinct roles these elements play in natural communities.

## Abstract (web summary)

Here, the authors report a large-scale comparative analysis of <30,000 Diversity-Generating Retroelements (DGRs) across ~9000 metagenomes (representing diverse taxa and biomes), to identify patterns in terms of prevalence and activity. Combined with examination of longitudinal data on <100 metagenomes part of time series, they demonstrate that DGRs are broadly and consistently active, implying an important role in microbiota ecology and evolution.

## Introduction

**Fig. 1:**
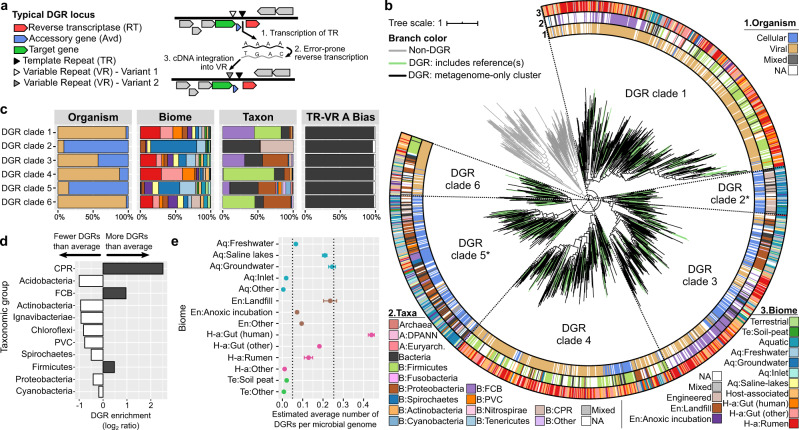
Distribution of DGR diversity across organisms, biomes, and taxa. **a** Schematic representation of the DGR mutagenic retrohoming process. The main components of a DGR are highlighted in colors, and the three main steps of the process are indicated directly on the diagram. TR, Template repeat/region. VR, Variable repeat/region. **b** Phylogeny of DGR and non-DGR reverse transcriptases (RT). RT protein sequences were first grouped into “RT clusters”, and a representative for each cluster was included in the tree building process (see “Methods”). Branches are colored according to the type of RT in the corresponding cluster. All nodes with support <50% were collapsed. From inside to outside, the outer rings display the consensus genome type, taxonomic classification, and biome of each RT cluster. CPR: Candidate Phyla Radiation. DPANN *Diapherotrites*, *Parvarchaeota*, *Aenigmarchaeota*, *Nanoarchaeota*, *Nanohaloarchaeota*, FCB *Flavobacteria*, *Fibrobacteres*, *Chlorobi*, *Bacteroides,* PVC *Planctomycetes*, *Verrucomicrobia*, *Chlamydiae*, Aq aquatic, Te Terrestrial, En Engineered, H-a Host-associated. NA corresponds to cases for which the feature could not be estimated (see Supplementary Fig. 2). **c** Distribution of each feature at the RT OTU level across DGR clades. The colors used in the bar chart are identical to panel (**b**), and NA values were not included. **d** Enrichment of DGR-encoding genomes across taxa. The total number of genomes observed across metagenome assemblies was calculated based on single-copy marker genes (see “Methods”), and an average frequency of DGR was derived from the entire dataset. A frequency of DGR detection per genome was then calculated for each taxonomic group and compared to the overall frequency to derive log_2_ enrichment ratios. All log_2_-ratios presented in the figure are statistically significant (Chi-square test of independence corrected *p*-value < 1E−10) except for the *Cyanobacteria* group (*p*-value = 0.21). **e** DGR enrichment across biomes. For each biome, a linear regression was computed between the estimated total number of genomes and the number of DGRs detected in each metagenome (see Supplementary Fig. 5). The regression slope was then considered as an estimation of the average number of DGR per genome and is displayed here with error bars representing the standard error of the slope estimation. Cutoffs of 0.05 and 0.25 DGRs per genome are highlighted with vertical dashed lines. For these calculations, viral and low complexity metagenomes were excluded (see Methods and Supplementary Data 1). Dots are colored according to the biome type (blue: aquatic, brown: engineered, pink: host-associated, green: terrestrial).

Diversity-generating retroelements (DGRs) are genetic elements that can produce a large number of mutations in a specific region of a target gene through error-prone reverse transcription^1,2^. The first DGR identified induces hypervariation in a structural protein responsible for host recognition and attachment of bacteriophage BPP-1^3^. Other examples of DGRs have been subsequently characterized, with the best-studied instances in *Legionella* and *Treponema* that target surface-displayed proteins^4,5^. All currently known DGRs seem to use the same molecular mechanism, known as mutagenic retrohoming, to generate hypervariation in the target protein^1,6,7^. Mechanistically, a DGR requires three main components: a reverse transcriptase (RT); a template region (TR), which in most cases is intergenic; and a variable region (VR) that is nearly identical to the TR and located within the coding sequence of the target protein (Fig. 1a). The DGR-encoded RT uses a primary TR transcript as the template for error-prone reverse transcription. Our current understanding is that DGR RTs are strongly promiscuous at template adenines, leading to the incorporation of dATP, dGTP, and dCTP in roughly equal proportions to the templated dTTP. The resulting sequence variant, i.e., TR-cDNA, is then integrated into the protein-coding gene, replacing the original VR sequence, through a yet-undefined homing mechanism^6,7^.

Building on the handful of well-characterized DGRs, recent studies have sought to explore DGR diversity by mining genomic data for DGR-like RT genes located next to imperfect repeats with mismatches opposing adenine positions. This approach was successfully applied to both draft genomes^2,8,9^ and metagenome assemblies^10–17^. Collectively, these studies identified ~1500 DGRs and suggested that DGRs are present in diverse environments ranging from the human gut to deep-sea sediments and terrestrial groundwater^10,12,13^. DGRs were also associated with a broad range of genomes including uncultivated bacteria from the candidate phyla radiation (CPR) and several archaea^12,13^, as well as human gut phages^15^.

While DGRs are now broadly recognized as important diversification mechanisms in microbes, their specific activity and role across organisms and biomes remain elusive. Specifically, ecological and evolutionary drivers of targeted hypermutation are currently unknown due to the lack of a global contextualized map of DGRs. Similarly, predicting the potential role of individual DGRs is currently challenging because the vast majority of putative targets are functionally uncharacterized. Therefore, it remains unclear for which proteins, functions, organisms, or environments this type of targeted hyper-diversification constitutes a selective advantage.

Here we analyze 31,007 DGRs identified from public metagenomes and metatranscriptomes to obtain a holistic view of DGR diversity and their spatial and temporal dynamics. We leverage this uniquely comprehensive DGR collection to (i) evaluate the global ecology and evolution of DGRs across viral and cellular genomes, (ii) characterize the functional diversity and molecular constraints of DGR targets, and (iii) infer temporal patterns of DGR activity across organisms and biomes. Taken together, these analyses reveal how DGRs are frequently transferred between genomes, yet clearly restricted to specific ecological niches, within which they likely impact both viral and microbial dynamics by driving sustained amino acid-level diversification of their target domains.

## Results

### Large-scale metagenome mining uncovers an extensive diversity of DGRs

To identify candidate DGRs, we searched for RT genes found within 1 kb of an imperfect repeat and used phylogenetic placement and mismatch patterns to identify false-positive detections (see “Methods”). We applied this approach to 81,404 public genomes and 9467 public metagenomes, representing 163 environment types, to obtain a global view of DGR diversity (Supplementary Data 1). In the analyzed genomes, we detected a total of 1314 DGRs, comparable in number and diversity to those identified in previous mining of genome databases^2,13^. Meanwhile, we detected 31,007 DGRs in public metagenomes, a ~15-fold increase compared to the total number of DGRs previously reported^10–17^. Overall, DGRs were detected from ≥1500 bacterial and archaeal genera and ≥90 environment types (Supplementary Data 2, Supplementary Note 1). Notably, because of the fragmented nature of metagenome assemblies (median size of DGR-encoding contigs: 9584 bp), our approach would not detect DGRs acting on remote targets^9,13^, i.e., DGR for which the VR is not located next to the RT. This dataset thus likely still underestimates the true DGR diversity.

Next, we used average amino acid identity (AAI) to group RT sequences, first into 13,415 OTUs (≥95% AAI), then into 1318 clusters (≥50% AAI, Supplementary Fig. 1, Supplementary Data 3 and 4). Members of each OTU and cluster were associated with the consistent genome (i.e., viral vs cellular), taxa, and biome types, suggesting that these groupings represent cohesively and mostly vertically inherited DGR evolutionary units (Supplementary Note 2, Supplementary Fig. 2). To obtain an overview of global DGR diversity, a phylogenetic tree was then built including a representative of each RT cluster along with other RT genes such as group II introns, retrons, and uncharacterized RTs (Fig. 1b). DGRs formed a monophyletic clade separated from all other types of RTs, supporting a single evolutionary origin for these elements, as previously hypothesized^2,18^. Overall, 75% of clusters were composed exclusively of metagenome-derived DGR sequences. Sequences from our survey spanned across an almost six-times larger phylogenetic diversity (573%) compared to previously known DGRs^2^, highlighting the significant contribution of metagenome and metatranscriptome assemblies to the exploration of DGR sequence space.

### DGRs dispersion is strongly constrained and reflected in cohesive lineage partitioning

Mapping the organism type (viral or cellular), taxonomy, and biome from which each DGR cluster was derived onto the tree suggested that the global DGR diversity could be divided into six main clades (DGR clades 1–6, Fig. 1b, c, Supplementary Note 3). Three clades (DGR clades 1, 4, and 6) are composed of DGRs identified almost exclusively in viruses, predominantly phages that are predicted to infect abundant gut bacteria belonging to the *Firmicutes, Bacteroidetes*, or *Proteobacteria* (DGR clades 1, 4, and 6, Fig. 1c, Supplementary Fig. 3). Clades 2 and 5 are almost entirely composed of cellular-encoded DGRs, mostly from aquatic biomes, and either restricted to the *Patescibacteria*, also referred to as the CPR (DGR clade 2), or affiliated to diverse phyla including *Proteobacteria* and *Bacteroidetes* (DGR clade 5). Finally, clade 3 includes a nearly even mix of virus- and cell-derived DGRs that are mostly associated with *Proteobacteria* and *Bacteroidetes*.

While mutation bias, i.e., mismatches opposing adenine positions, was not used here as a criterion for DGR detection, alignments between template repeat (TR) and variable repeat (VR) overwhelmingly displayed ≥75% of mismatches facing adenine residues in the TR across all clades (Fig. 1c). After manual inspection of outliers (Supplementary Note 4), we identified only 7 clusters of seemingly genuine DGRs with <75% of mismatches facing adenine residues (Supplementary Fig. 4, Supplementary Data 4). This is consistent with previous comparative genomics and biochemical studies^6,7^ and confirms that the mutation bias towards adenine is an intrinsic feature of DGR RTs. The monophyly in the RT tree and universality of the adenine mutation bias combined suggests a single origin for all currently known DGRs, followed by sporadic transfers across organisms and biomes leading to the six main clades observed here. This evolutionary scenario is also consistent with the hypothesis of DGRs being initially encoded on mobile genetic elements, which could spread DGRs across many taxa and ecosystems.

Across the 9467 metagenomes we examined, several taxa and biomes were clearly enriched in DGRs. DGRs were significantly more common (*p*-value < 10^−16^) in members of the CPR, *Firmicutes*, and flavobacteria-bacteroidetes-chlorobi (FCB) groups (Fig. 1d). Similarly, we observed a significantly higher rate of DGR detection per genome in samples from several environments, including the human gut, saline lakes, landfills, and groundwater reservoirs (Fig. 1e, Supplementary Fig. 5). Phylogenetic logistic regression further confirmed that both phylogeny and ecology drive the distribution of DGRs (Supplementary Note 5). DGRs are associated with specific monophyletic clades in both cellular and viral genome trees. After accounting for this phylogenetic signal, viral-encoded DGRs are still significantly positively correlated with specific biomes, while cellular-encoded DGRs are only negatively correlated with a single biome (Supplementary Table 1). Taken together, these results point towards a long and complex DGR evolutionary history, with DGRs able to transfer between phylogenetically unrelated organisms, but only retained in specific niches in which targeted mutation would represent a selective advantage and/or in taxa able to support DGR mutagenic retrohoming. This hypothesis is supported by the broad yet uneven distribution of these elements across genomes and biomes.

### DGR targets share a conserved organization

**Fig. 2:**
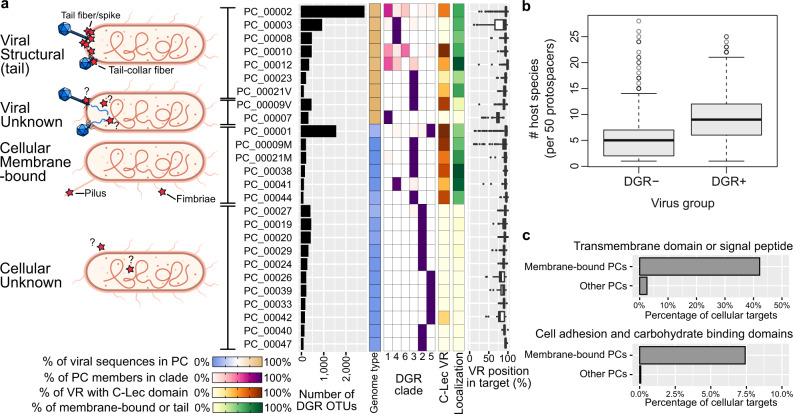
Diversity and major types of DGR targets. **a** Prevalence and sequence characteristics of the most abundant DGR target protein clusters (PCs). The 24 PCs listed here represent >92% of all identified DGR targets. These were divided into four major types (left panel; predicted localization of target proteins highlighted with red stars). Characteristics of each PC are indicated to the right, including a number of associated DGR RT OTUs, the relative proportion of viral-encoded vs. cellular-encoded DGRs (“Genome type”), distribution across DGR clades, percentage of PC member with a detected C-Lec fold around the VR region, percentage of PC members predicted as a tail structural protein (for viral targets) or membrane protein (for cellular targets), and relative position of the VR region within the target sequence. The boxplot lower and upper hinges correspond to the first and third quartiles, respectively, and the whiskers extend no further than ±1.5 times the interquartile range. For C-Lec fold VR and localization prediction data, only high-quality targets were considered (see “Methods”). For mixed PCs (PC_00009 and PC_00021), targets with an unknown origin were excluded. **b** Estimated host diversity for viral genomes encoding (DGR+, *n* = 822) or lacking DGRs (DGR−, *n* = 1182) matching at least 50 CRISPR spacers. The vertical axis shows the number of connected host species per 50 protospacers. The boxplot lower and upper hinges correspond to the first and third quartiles, respectively, and the whiskers extend no further than ±1.5 times the interquartile range. **c** Percentage of cellular targets with a predicted transmembrane domain (top) or one or more functional domain(s) associated with cell adhesion and/or carbohydrate-binding identified outside of the VR region (bottom). Target sequences are divided based on their PC membership into “membrane-bound” PCs or “other” PCs (see panel (**a**)).

To gain insight into the potential roles of identified DGRs, we next investigated the diversity of the 36,611 genes identified as putative DGR targets across genomes and metagenomes. As previously reported^2^, the majority (68%) of these targets could not be functionally annotated when individually compared to reference databases. However, de novo clustering revealed that most DGR targets (>92%) grouped into 24 protein clusters (PCs), which clearly partitioned by genome type and DGR clade (Fig. 2a, Supplementary Data 5, Supplementary Fig. 6, Supplementary Note 6).

Analysis of functional domains and residue conservation across PCs suggested a near-universal modular organization. Targets were typically multi-domain proteins, with the VR region found at the C-termini (Fig. 2a), which corresponds to the previous observations^2^. This positioning of VR regions at the C-termini of target proteins is likely due to the requirement of cis-acting DNA elements for retrohoming^19^, since only for C-terminal VRs can these cis-acting elements be intergenic and thus free from amino-acid coding constraints. While these C-terminal regions include DGR-variable residues, they were overall more conserved than the rest of the sequence across all PCs, likely due to structural constraints associated with DGR-induced hypervariation^20,21^ (Supplementary Fig. 7). Accordingly, whereas a range of folds was predicted for N-terminal domains, annotated VR-containing regions were systematically associated with C-type lectin (C-Lec) folds. Some rare VRs had previously been tentatively linked to Ig-like folds^2^, but a re-analysis of these sequences, leveraging the larger context from our expanded catalog, suggests these targets instead correspond to phage tail fibers containing Ig-like domain(s) next to an uncharacterized, non-Ig-like, VR domain (Supplementary Figs. 8 and 9, Supplementary Note 7). Since novel variants of C-Lec fold domains are still being discovered on a regular basis^20,22^, it is probable that other uncharacterized conserved domains overlapping VR regions represent new variants of the C-Lec fold domain family (Fig. 2a). Based on the distribution of corresponding target PCs, these novel C-Lec fold will most likely be associated with novel viruses and uncultivated bacteria (CPR), and archaea (Supplementary Fig. 9). The observed modularity of target proteins also suggests that intragenic recombination may occur for DGR targets, with the potential to fuse a wide range of independently folding domains to a C-terminal C-Lec-encoding region to produce a chimeric target ready for mutagenesis.

### DGR targets are primarily involved in virus-cell and cell-particle interactions

Given the near-universal modular organization of target proteins, putative functions were assigned based on the presence of conserved domains or sequence features identified outside of the C-terminal VR region. Within the 24 main target PCs, a majority of sequences (71%) did not display any significant sequence similarity to any known protein domain outside of the C-terminal VR region, even when using highly-sensitive annotation tools such as HHblits^23^. Hence, we instead classified targets into broad functional classes, namely “structural proteins” vs “unknown” for viral-encoded DGRs and “membrane-bound” proteins vs “unknown” for cellular-encoded DGRs, using non-similarity-based protein annotation approaches^24,25^ (Supplementary Note 8).

Viral targets from DGR Clades 1, 4, and 6 were mostly annotated as tail structure proteins, which are typically involved in host recognition and attachment (Fig. 2a). This includes the target protein from the original report of DGR-mediated mutation of phage tail fibers in *Bordetella* bacteriophages which has been shown to enable host switching^3^. We reasoned that hyper-mutation of host attachment proteins may broadly enable DGR-encoding viruses to access a larger diversity of host cells. We tested this hypothesis by connecting DGR- and non-DGR-encoding viruses to a comprehensive database of 6.7 million CRISPR spacers derived from 576,561 prokaryotic reference genomes^26^. This analysis revealed that DGR-encoding viruses are associated with a significantly larger diversity of hosts than non-DGR-encoding ones, even after controlling for several confounding variables (Fig. 2b, Supplementary Fig. 10, Supplementary Note 9). While these results suggest that DGR-mediated hypermutation enables phages to attach to and inject their genome into a broader range of host cells, it does not necessarily imply that the phages are then able to perform a successful and efficient replication cycle. Several host characteristics and defense mechanisms can lead to aborted infections^27^, and a number of additional adaptations will likely be required for these viruses to successfully infect any new taxon, even ones closely related to their original host^28^.

For cellular targets, most PCs contained at least one N-terminal transmembrane domain or signal peptide, along with functional domains involved in protein binding, carbohydrate-binding, and cell adhesion (Fig. 2a, c, Supplementary Data 5). This suggests that most of these targets are membrane-anchored proteins that bind extracellular substrates, possibly including particle aggregates, other microbial cells, or viral particles. Accordingly, metagenome-assembled genomes (MAGs) associated with the most prevalent of these targets (PC_00001) displayed a gene content and functional annotation consistent with a copiotrophic or particle-associated lifestyle (Supplementary Note 10). DGRs with PC_00001 targets were primarily detected in aquatic environments (Supplementary Fig. 9), although the frequency of DGR detection was highly variable between different aquatic biomes (Fig. 1e, Supplementary Fig. 5). Taken together, this suggests that the selective advantage provided by broad-scale particle binding, cell–cell attachment, or surface adherence may vary between environments. For instance, in the open ocean, random binding may not be advantageous as it could lead to elevated cell loss due to sinking particles^29^, which may explain why DGRs are rarely detected in these samples (Fig. 1). Importantly, however, cellular membrane proteins can also be used as host receptors by viruses, and some of these targets may thus be under DGR-driven diversification as part of a virus-host arms race^30^. Unlike most other cellular-encoded targets, the ones encoded by CPRs of clade 2 and archaea of clade 5 do not typically include any recognizable transmembrane or other conserved domain (Fig. 2c), as previously reported^12,13^. Whether this is due to functional domains that are not readily identified in these divergent genomes or because these proteins represent genuine non-membrane-bound DGR targets remains to be established. Overall, the large collection of DGR targets identified in this study provides additional and strong evidence that DGRs are primarily linked to cell-particle, cell–cell, and virus–cell interactions, and in some rare cases may be involved in microbial cell regulation^13^.

### DGRs are broadly active across organisms and biomes

**Fig. 3:**
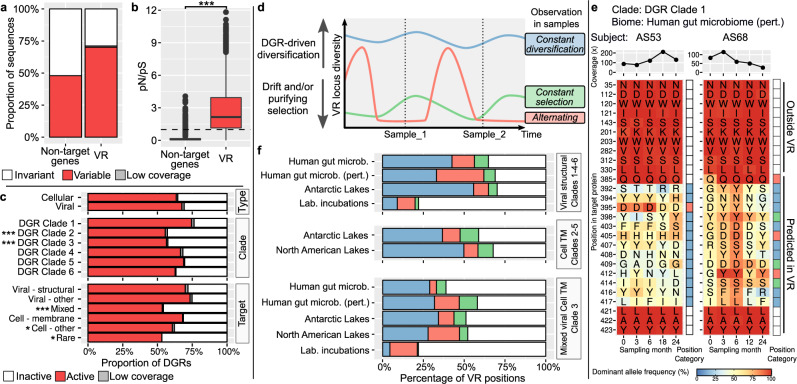
Diversity patterns associated with DGR target loci. **a** Proportion of genes with ≥1 SNV observed (synonymous or non-synonymous), for both non-target genes within 10 kb of a DGR RT (left) and VR loci (right). “Low coverage” category includes cases in which the coverage of the VR region was significantly lower than that of the surrounding genes, suggesting that the read recruitment may be only partial, and the population diversity in the VR cannot be reliably inferred (see Supplementary Fig. 11). **b** Distribution of pN/pS values for genes with ≥1 synonymous SNV, for both non-target genes and VR loci. A dashed line indicates pN/pS = 1. Boxplot lower and upper hinges correspond to the first and third quartiles, whiskers extend no further than ±1.5 times the interquartile range. pN/pS distributions for non-target genes and VR loci were compared using a Kruskal–Wallis test (***p-value < 2.2E−16 and Cohen’s d effect size >1.7). **c** Proportion of DGRs estimated as “active” vs. “inactive” based on enrichment of VR loci in non-synonymous SNVs compared to surrounding genes, across different DGR classifications. Groups with a significantly lower proportion of active sequences (Chi-squared test of independence) are highlighted with star symbols (Bonferroni-corrected *p*-values: *<1E−03, **<1E−05, ***<1E−10). **d** Schematic representation of the two competing forces exerted on VR loci: purifying selection and DGR diversification. Three examples of possible DGR activity levels are indicated in color, with the resulting observations across a time series (“Sample_1” and “Sample_2”) summarized in the right column. **e** Example of diversity and changes observed for one DGR target across two-time series datasets. For each position, the corresponding amino acid is indicated in the main heatmap with its frequency within the population indicated in color. The right panel indicates the category of the position, colored as in panel (**d**), based on within-sample entropy, between-samples cosine distances, and the number of amino acid changes in the time series (see Supplementary Note 12). The top panel indicates the median coverage of all positions in each sample. For reference purposes, ten random positions from the same protein outside of the predicted VR are included. **f** Distribution of VR positions into “activity” categories (colored as in panel (**d**)) across different biomes and clades. Cases with <50% of variable positions and <5% of amino acid changes were considered as “low DGR activity” and colored in white. Only groups for which ≥10 DGRs were available are included.

Next, we evaluated the population diversity at DGR loci across taxa and ecosystems. To that end, we analyzed single-nucleotide and amino acid variants^31,32^ for 6901 DGRs with ≥10 kb genomic context and ≥20× coverage (see “Methods”). Overall, single-nucleotide variants (SNVs) could be detected for 70.1% of the VR loci (Fig. 3a, Supplementary Fig. 11, Supplementary Note 11). When SNVs were detected, VR loci were strongly enriched in non-synonymous SNVs. We first evaluated this through the ratio of non-synonymous to synonymous polymorphism rates (pN/pS^33^): while nearly all non-target genes displayed pN/pS < 1 consistent with a purifying selection, >80% of VR loci with ≥ 1 SNV(s) displayed pN/pS ratios > 1, indicating a strong enrichment in non-synonymous mutations (Fig. 3b). Since pN/pS cannot be calculated in the absence of synonymous SNV, and 36% of VR loci displayed exclusively non-synonymous SNVs, we opted to directly estimate the enrichment of VR loci in non-synonymous SNVs as a marker for recent DGR activity (see “Methods” and Supplementary Note 11). We reasoned that when VR loci displayed a significantly higher non-synonymous SNV density than their neighbor genes, this was the result of a recent and/or ongoing DGR-driven mutagenesis.

For all DGR groups, 50–75% of DGRs showed signs of recent activity (Fig. 3c). Viral-associated DGRs were linked to the highest activity level, while members of DGR clades 2 and 3 displayed a significantly lower-than-average activity level (Supplementary Fig. 12). However, the strong purifying selection that would reduce population diversity may mask DGR activity in these single-sample variant analyses, i.e., active DGRs may generate new variants that would almost instantaneously be purged from the population and thus evade detection.

### DGR activity drives frequent changes in target residues

Given widespread DGR activity, most VR loci can be expected to evolve under two antagonistic forces: DGR diversification and purifying selection. The relationship between these forces can be examined from time-series data so long as the adaptive value of different variants fluctuates over time (Fig. 3d, Supplementary Fig. 13, Supplementary Note 12). Specifically, we hypothesized DGR diversification to lead to high population diversity within each sample and changes in dominant allele between time points, while purifying selection would reduce population diversity within each sample. For alternating phases of diversification and purifying selection, we expect to observe a low population diversity within each sample but changes in the dominant allele between samples (Supplementary Fig. 14).

To test this hypothesis and to shed light on the balance between DGR diversification and purifying selection in nature, we analyzed the subset of DGRs found across metagenomic time series. We identified 130 longitudinal data sets containing 563 DGRs amenable to analysis, i.e., having ≥10 kb genomic context, with coverage ≥10×, and detected at ≥2 time-points (Supplementary Data 6). Overall, a majority of predicted VR positions in these DGRs showed high diversity associated with frequent amino acid replacement, suggesting a high DGR activity overpowering purifying selection (Fig. 3e, f). This pattern was consistent across natural biomes but absent for in vitro microbiomes, i.e., laboratory incubations. There, both the observed diversity and amino acid change frequency were much lower for all types of DGRs, probably due to the population bottlenecks^34,35^, lack of various environmental stress, and/or shorter time frames of these experiments (Fig. 3f). Overall, viral-encoded DGRs in clades 1, 4, and 6, targeting mostly structural proteins, systematically displayed a higher rate of amino acid turnover than DGRs of the cellular-encoded clades 2 and 5 or from “mixed” clade 3 from the same environments (Fig. 3f, Supplementary Fig. 15). Extrapolating from the average mutation rates observed here, we conservatively estimated that DGR-driven mutations would be responsible for 6–16% of all amino acid changes in an average viral genome, even though DGRs target only ~0.1% of amino acid residues (see Supplementary Note 13). In addition, we observed a higher level of DGR activity in human gut samples from individuals following a 12-month weight-loss program^36^ compared to “unperturbed” gut microbiome samples from the Human Microbiome Project^37^, with a more pronounced increase in activity for cellular-encoded clade 3 DGRs (Fig. 3f, Supplementary Fig. 16). Taken together, this suggests that DGRs drive more steady changes in viral structural proteins through time compared to non-structural or cellular targets, and some of the latter may be more associated with adaptation during stress episodes. Whether this is due to stronger control of DGR RT activity or a stronger selection exerted on targets outside of stress episodes remains to be determined.

## Discussion

The extensive comparative analysis of metagenome-derived DGRs presented here highlights DGRs as a fundamental component of microbial and viral genome evolution. The near-universal conservation of the adenine mutation bias and the C-Lec fold in target proteins suggests that DGR RTs are mechanistically constrained in the type and location of mutations they can generate and, reciprocally, that C-Lec folds have a seemingly unique ability to accommodate massive sequence variation^20^. The strong DGR enrichment observed in select biomes and taxa likely reflect specific ecological conditions and lifestyles for which hypermutation is advantageous. For instance, in human gut microbiomes, the combination of high resource availability and frequent infections by a broad diversity of phages is expected to favor resistance through cell wall modification^38^. This would in turn select for DGR-encoding viruses, which would leverage hypervariation of host recognition proteins such as tail fibers to bypass these host resistances^39^, and possibly expand their range of potential hosts in the process. Finally, the widespread and seemingly constant DGR activity suggests that these elements are mostly used to maintain a high population diversity at target loci rather than only being triggered in case of extreme stress. Overall, our global analysis of DGR diversity and activity indicates that DGRs likely shaped long-term microbe-microbe and virus-host interactions in multiple taxa and biomes; that they drive the diversity and evolution of key components of viral particles and microbial cells envelopes; and that they may represent a fundamental mechanism by which viruses and cellular microorganisms adapt and respond to an ever-changing environment.

## Methods

### Collection and annotation of reverse-transcriptase (RT) and DGR reference sets

Reference sequences of RTs (DGRs and non-DGR) were collected from ref. ^18^. Additional DGR references were obtained from ref. ^2^. For the latter, corresponding TR/VR and target sequences were extracted from the supplementary html and fasta files (respectively) provided. Taxonomic classification of reference sequences was derived from the NCBI database based on the genome identifier provided for each DGR. For DGRs not taxonomically classified as viruses, the genome sequence was downloaded from NCBI GenBank and VirSorter was used to identify whether the DGR RTs were encoded in an integrated provirus or metagenomic viral contig.

### Detection of DGRs in (meta)genomes

The overall detection pipeline consisted of three main steps: (i) identification of RT based on matches to HMM profiles using hmmsearch v3.2.1^40^, (ii) detection of repeats around the candidate RT using blastn v2.9.0+ ^41^ with option -word_size 8 -dust no -gapopen 6 -gapextend 2, and (iii) selection of putative DGRs, TRs, and target genes based on repeat patterns and RT length. Importantly, unlike existing tools^42^, this detection was agnostic to the variation between the repeats, because it did not require mismatches between repeats to be associated with adenine residues. The input sequences for this detection pipeline were (i) all genes predicted from IMG public genomes, and (ii) genes predicted on contigs ≥1 kb and encoding ≥2 genes from IMG public metagenomes (Supplementary Data 1). The former (i.e., IMG public genomes) were used to complete the reference set of DGRs already collected from the literature. These sequences were included in the DGR catalog studied here (see Supplementary Data 1, Dataset Type “Genome”), but were not the subject of any targeted analysis. The latter (i.e., sequences from IMG public metagenomes) formed the bulk (96%) of the dataset analyzed in this study. Notably, while IMG public metagenomes do not include the entirety of publicly available metagenomes, this database was selected because it includes datasets from a very broad range of environments and taxa, enabling a global survey of DGR diversity.

Successive rounds of the detection pipeline were performed as follows. Candidate DGRs were first detected based on matches to Pfam reverse-transcriptase domains (PF00078, PF07727, PF13456, and PF13655), with a score of ≥20 for genomes and ≥30 for metagenomes^43^. Candidates with an RT sequence length of 250–550 amino acids and an imperfect repeat detected within 20 kb of the RT in 5′ or 3′ (blastn hit ≥50 bp with <99% nucleotide identity), with one of the repeats within ≤1 kb of the 5′ or 3′ end of the RT gene, were selected as putative DGRs. Sequences obtained from isolates were clustered with literature-derived references to remove redundancy (cd-hit^44^ v4.8.1, ≥95% AAI). All candidate DGRs were included in a phylogenetic tree along with DGR and non-DGR references, based on multiple alignments of RT amino acid sequences obtained with mafft^45^ v7.407 using iterative refinement (“einsi”), and built with FastTree^46^ v2 using the WAG substitution model. Since known DGRs formed a large monophyletic clade in this phylogeny, new sequences that branched within this clade were assumed to be likely DGR, regardless of the characteristics of their predicted TR and VR sequences, i.e., whether or not the TR was intergenic and the mismatches between TR and VR reflected an A mutation bias. Representatives of each novel RT group that branched outside of the known DGR clade were manually inspected to evaluate whether these could represent novel DGRs. In all such cases, we observed that the predicted TR and VR were found in genome regions with high repeat content, often next to insertion sequences or transposases, TR and VR were typically either both predicted as part of a cds or both intergenic, and TR-VR alignments typically did not show any mutational bias. We reasoned that these RTs were unlikely to represent new DGR elements, and to enable automatic filtering of these sequences branching outside of the main DGR clade, all those for which <75% of the mismatches between repeats were associated with A residues were discarded. All retained candidate DGRs were used to generate six new HMM profiles representing the main clades in the RT tree, using multiple alignments built with Muscle^47^ v3.8 and the hmmbuild tool from HMMER^40^ v3.2.1, with default parameters.

A second round of search was conducted on the same input dataset by using these new DGR RT HMM profiles instead of Pfam HMM profiles in the initial search (score ≥ 50 and *e*-value ≤ 1E−05). Putative DGRs were then selected as in the first round, except for the RT sequence length which was extended to range from 150 to 650 amino acids. After manual inspection and removal of false-positive detections based on a phylogeny (as in the first round), another set of 4 HMM profiles were constructing. These new HMM profiles were used in a third and last round of search, which did not yield any new plausible candidate DGR, detecting only seven previously undetected sequences that were all identified as likely false positives. The protein sequences of all DGR RTs are provided as Supplementary Data 7, the protein sequences of the predicted targets as Supplementary Data 8, and the nucleotide sequences of the TR-VR pairs as Supplementary Data 9.

### Selection and annotation of reference genomes and metagenome sequences

For additional DGRs identified from IMG genomes, taxonomic classification was derived from the IMG taxonomy database. VirSorter^48^ v1.0.5 was used to identify which of these DGRs were encoded in proviruses: predictions of categories 1, 2, 4, and 5 were considered as viral, while predictions of categories 3 and 6 were provisionally listed as “putative viral” for the RT OTU-level aggregation (see below).

For DGRs detected on metagenome contigs, the taxonomic classification of DGR-encoding contigs ≥3 kb were derived from the automatic taxonomic annotation provided by IMG, based on majority ruling from gene-level best blast affiliations, i.e., each contig is affiliated based on individual gene affiliation up to the rank at which there is no majority affiliation anymore^49^. For contigs <3 kb, taxonomic classification was set as “unclassified” because there are not enough predicted genes beyond the RT and DGR target on these contigs for a robust classification using the majority rule approach. Viral origin was predicted using a combination of VirSorter^48^ v1.0.5 (as for genomes), the Earth’s Virome pipeline^50^, and the inovirus detector pipeline^51^. All sequences identified as viral with the Earth’s Virome pipeline, the inovirus detector pipeline, or VirSorter categories 1, 2, 4, and 5 were considered “viral”, while sequences only predicted with VirSorter as categories 3 or 6 were listed as “putative viral” for the RT OTU-level aggregation (see below). For sequences not identified as viral by any pipeline, contigs ≥10 kb were considered as “cellular”, while contigs <10 kb were considered as “unknown”, based on previous benchmarks of viral sequence detection tools^48,50^. For contigs identified as viral, host taxonomic classification was determined as follows. For contigs ≥10 kb, IMG blast-based taxonomy was considered as the predicted host taxonomy^52^. All sequences predicted as viral were also compared to IMG CRISPR spacer database^53^ using blastn^41^ with options—dust no and—word_size 7. Hits between viral sequences and CRISPR spacers with 0 or 1 mismatch over the entire spacer length were selected and used to infer host taxonomic classification of the corresponding viral sequence. In the rare cases where IMG and CRISPR match taxonomic affiliations were inconsistent, IMG taxonomy was used. Viral contigs were also classified using vContact2 v0.9.10^54^, to obtain a taxonomic affiliation of the virus itself (instead of the host) at the genus rank. vContact2 was run with the “diamond” option to generate the PCs, clustering of VCs with cluster_one, and the reference database “ProkaryoticViralRefSeq94-Merged”, all other parameters left as default. Metagenome-derived DGRs were also associated with a biome type based on the original sample classification available in the Gold database^55^ (Supplementary Data 1).

In all analyses, the taxonomic classification used was the microbial one (i.e., “host” classification for viral contigs) at the domain and phylum ranks or equivalents. Members of the Candidatus Phyla Radiation (for bacteria) and DPANN (for archaea) were gathered in “Bacteria:CPR” and “Archaea:DPANN” groups (respectively) based on the supergroup classification proposed in ref. ^56^. Similarly, genomes classified as Bacteroidetes, Chlorobi, Cloacimonetes, Fibrobacteres, and Marinimicrobia were gathered in a “Bacteria:FCB” group, and genomes classified as Omnitrophica, Chlamydiae, Lentisphaerae, Planctomycetes, and Verrucomicrobia in a “Bacteria:PVC” group.

### Clustering and annotation of DGR based on RTs

The global DGR collection was clustered based on RT sequences, along with non-DGR RTs, representing a total of 33,342 RT sequences: 655 non-DGR RTs, 1680 “reference” DGR RTs either from literature or from IMG genomes, and 31,007 from IMG metagenomes. These sequences were first clustered into “RT-OTUs” at 95% AAI using cd-hit^44^ v4.8.1. Next, the representatives (longest sequences) from each RT-OTU were collected and compared all-vs-all using blastp^41^ v2.9.0+. Blast hits with *e*-value < 0.001 and with AAI ≥50% (based on the whole query length) were used as input to an MCL clustering (v14-137) with inflation value 2.0 and AAI percentage as edge weight^57^. The groups provided by MCL are designated as “RT-Clusters”.

RT-OTUs and RT-Clusters were associated with a genome type (viral or cellular), taxonomic classification, biome classification, and target gene (see below) as follows. Because DGRs were detected across >1500 different bacterial, archaeal, and viral genera, and >90 different environment types, we opted to conduct global analyses at a coarse level, i.e., phylum-level for taxonomy and broad ecosystem types (e.g., “Aquatic:Freshwater”, “Host-associated:Rumen”) for biomes. Taxonomic classification of RT-OTUs was based on RT-OTU member affiliations using a majority rule, and an LCA if the top two affiliations had an identical number of members. A similar approach was used for the biome classification and the target PC, i.e., majority rule and LCA in case of a tie. For genome type, RT-OTUs with all members unknown were considered “unknown”, RT-OTUs with at least 1 “viral” or “putative viral” member and no cellular were considered “viral”, while others were considered “viral” or “cellular” based on a majority rule between viral and cellular members (if tied, the RT-OTU is considered as “unknown”). A consensus bias vector, representing the frequency of individual A, T, C, and G nucleotides in the TR for positions with mismatch, was also calculated by averaging the bias vector of RT-OTU members. For this average, we discarded cases in which the RT was found within 500 bp of the contig edge of the target gene was found within 200 bp of the contig edge, and the bias vector had an atypical A frequency <70%, as these likely represent misprediction of the TR/VR and/or target (based on manual inspection of these contigs). Similarly, in cases where RT-OTUs included both members with “typical” and “atypical” bias vectors, i.e., A frequency ≥70% and <70% respectively, the average vector for the RT-OTUs was calculated only with the “typical” ones. For RT-Clusters, a 2/3rd majority rule was applied based on the annotation of the RT-Cluster members. For multi-level data (taxonomy, biome, and target PC), the 2/3rd majority rule was applied first to the first level, then to the second level. In cases for which the majority value was found in less than 2/3rd of the RT-Cluster, the value was set as “unknown”.

### Enrichments of DGRs across taxa and biomes

Two different approaches were used to evaluate potential enrichment in DGRs of specific taxa and/or biomes. To link DGRs with specific taxa, the number of genomes affiliated to each taxon (at the same rank as for the DGR, see above) was estimated for each metagenome based on a list of 139 single-copy marker genes^58^. Briefly, for each metagenome, the total number of genomes for a taxon was estimated as the median number of single-copy marker genes affiliated to this taxon, similar to the estimation performed in Anvi’o^59^. For each taxon, an enrichment in DGRs was calculated as the log_2_ ratio between the frequency at which DGRs were observed in this taxon (i.e., total number of DGR OTUs observed for this taxon divided by the total estimated number of genomes for this taxon across all metagenomes) and the average frequency of detection of DGRs across all taxa (i.e., total number of DGR OTUs divided by total estimated number of genomes across all metagenomes). The statistical significance of these differences in DGR frequency was evaluated using a Chi-square test of independence (prop.test function in R v3.6.1 on 2 × 2 contingency table for each taxon).

For biomes, the same set of single-copy marker genes were used to estimate the total number of microbial genomes in each metagenome. For each biome group (see above), a linear regression model was then fitted using the number of genomes as a predictor for the number of DGRs. For statistically significant fits (*p*-value <1E−4), an estimated number of DGRs per genome was derived based on the regression coefficient. Metagenomes with ≥40% of contigs ≥10 kb identified as viral were excluded from this analysis as they likely derive from samples strongly enriched in viral genomes, for which the count of microbial genomes will not be reliable.

### Clustering and functional annotation of predicted target genes

For each DGR RT, target genes were identified by comparing the predicted VR repeat to CDS predictions available in the IMG database. If multiple VR repeats were detected for a single DGR RT, the one with the highest A mutation bias or, if tied, the closest to the DGR RT on the contig, was considered as the primary target. Genes associated with other VR repeats were then included as “secondary” targets if the VR repeat was associated with a plausible A mutation bias (≥75% of mismatches on A positions in the TR).

For de novo clustering of predicted targets, high-quality target sequences were first selected as genes longer than 300 nucleotides and not within 50 bp of the edge of the contig, i.e., less likely to represent partial genes. These high-quality target genes were clustered at 99% AAI using cd-hit^44^ v4.8.1, then clustered in a two-step process as in ref. ^51^. Briefly, sequences are first clustered using MCL v14-137^57^ from an all-vs-all blastp, using blast score as edge weight and an inflation value of 2.0, then HMM profiles were built for these clusters and HHsearch^23^ v3.1.0 was used to identify similarities between clusters. This led to the definition of “superclusters” (i.e., clusters of clusters) based on a single-linkage clustering using similarities of ≥90% probability of ≥50% of the profile length or ≥99% over ≥20% of the profile length and 100 positions. Target sequences that were initially discarded because shorter than 300 nucleotides and/or within 50 bp of the contig edge were then mapped to these superclusters using hmmsearch^40^ v3.2.1, with each sequence affiliated to the cluster with the highest score if ≥30.

This two-step clustering pipeline was further evaluated on reference genomes to verify that it was not too sensitive and did not over-cluster either microbial or phage proteins. For this benchmark on cellular genomes, 25,000 protein sequences were randomly selected among all predicted cds in DGR-encoding IMG genomes (genome list provided in Supplementary Data 5). Two clustering analyses were performed: one including the 25,000 proteins, and one including a random subset of 15,599 sequences to match the exact input size of the DGR target clustering. For benchmarking viral protein clustering, 24,753 predicted cds from 200 *Caudovirales* genomes randomly selected from NCBI Viral RefSeq v201^60^ (genome list provided in Supplementary Data 5) were gathered and clustered using the same pipeline, including a separate clustering with a 15,599 sequences subsample. The different clustering results were compared by evaluating the number of sequences gathered in the 20 largest clusters (Supplementary Fig. 6) and by calculating Shannon’s Entropy index on each set of clusters using custom Perl scripts. Further, the functional annotation of individual PC members for both benchmark datasets is presented in Supplementary Data 5, to confirm that PCs obtained with this pipeline include protein sequences with similar functions.

Functional annotation of superclusters was obtained from analysis of the supercluster multiple sequence alignment and from the annotation of individual members. For the former, multiple sequence alignments were built using Muscle^47^ v3.8 after dereplicating the supercluster sequences at 90% AAI using cd-hit^44^ v4.8.1. These alignments were then used as input in HHblits^23^ which compared the alignments to the Pdb70 v190918, Pfam v32, and SCOPe70 v1.75 databases (database package downloaded in Feb. 2019 from the HH-Suite website). Each target sequence was also annotated the same way, using a direct hhblits^23^ comparison to the same Pdb70 v190918^61^, Pfam v32^43^, and SCOPe70 v1.75^62^ databases, and using an hmmsearch^40^ comparison to the Pfam v31 database^43^. Annotations were derived from hits with a score of ≥50 in hmmsearch or ≥90% probability in hhblits, except for hits overlapping the prediction VR region for which these cutoffs were lowered to ≥30 on score and ≥80% probability, in order to enable the identification of distantly related C-lectin folds. In addition, individual target sequences were also searched for transmembrane domains and signal peptides using TMHMM^63^ v2.0c (default parameters) and SignalP^64^ v4.1 (score D ≥ Dmaxcut), and searched for potential *Caudovirales* structural proteins (capsid or tail proteins) using DeepCapTail v3038c4d^24^ (version downloaded Jan. 2020) and PhANNs v1.0.0^25^ with thresholds of ≥0.9 and ≥0.2 on the score, respectively. The same clustering and annotation pipeline were applied to predicted cds from NCBI RefSeq *Caudovirales* genomes, after having dereplicated these protein sequences at 99% using cd-hit^44^ v4.8.1 (*n* = 250,209 proteins), in order to evaluate the functional annotation across all *Caudovirales* of (i) sequences containing an Ig-like domain (ii) sequences predicted as “capsid” or “tail” via DeepCapTail (see Supplementary Note 8).

A prediction of 3D structure was computed for selected target sequences using I-TASSER^65^ 5.1, using default reference libraries and 25 h-long simulations. The average conservation of residues in target clusters was based on the multiple alignments generated for cluster annotation (see above). For comparing conservation within and outside of the VR regions, a predicted “extended” average VR region was defined by adding 200 residues upstream and 20 residues downstream of VR regions predicted on individual sequences. The size of this “extended” VR regions was defined based on the coordinates of predicted VRs and surrounding conserved C-Lec fold on reference DGRs.

The association between RT, TR-VR, and target protein sequences was evaluated as follows. Predicted TR sequences associated with high-quality targets and typical mutational bias (see above) were compared using all-vs-all blastn^41^ v2.9.0+ with options adapted for short sequences (“-dust no -word_size 7”). The global nucleotide identity between two TR sequences was then calculated based on the number of identical residues in the best blast hit compared to the length of the shortest TR sequence of the pair.

### DGRs identified in genome bins

When available, automatically-generated genome bins (“GEM dataset”) were searched for DGR-encoding contigs. Briefly, genome bins were automatically generated for public metagenomes on IMG, using metabat^66^ v0.32.4 for binning with a 3000 bp minimum contig cutoff, contig coverage information, and parameter “-superspecific” for maximum specificity, checkM^67^ v1 for quality estimation, and gtdb-tk^68^ v0.3 for taxonomic assignment^26^. Only medium- and high-quality bins according to the MIMAG^69^ criteria were included. DGRs encoded on contigs identified as entirely viral were not considered in this process, since previous studies have indicated that these contigs are often binned incorrectly^26^. Overall, 13,180 MAGs were searched, and 1509 were found to include at least 1 DGR locus. For metagenomes including at least 1 genome bin with a DGR-encoding contig and at least 10 genome bins, the relative abundance rank of each genome bin was determined as follows: for all MQ and HQ genome bins identified in the metagenome, the bin coverage was estimated as the median coverage of all contigs. The genome bins were then ordered based on this median coverage of contigs to determine the rank(s) of genome bin(s) encoding DGRs.

The diversity of DGR-encoding genomes identified in human gut samples was evaluated through an RNA polymerase B (RpoB) tree. RpoB protein sequences were first identified in isolate genomes and genome bins associated with human gut and encoding a DGR of clade 1, 4, or 6, based on significant hits to the pfam domain PF04563 (hmmsearch score ≥50). Multiple alignments were then built with MAFFT^45^ v7.407 using default parameters, automatically trimmed using TrimAl^70^ v1.4.rev15 with the -gappyout option, and used as input to build a tree with IQ-Tree^71^ v1.5.5 with built-in model selection (optimal model suggested: LG + R6).

The gene content of DGR-encoding genomes was evaluated based on metagenome bins as follows. For each metagenome including at least one Clade 5 DGR in a genome bin, the number of proteins affiliated to each COG in each MQ or HQ bin was tallied. The number of proteins associated with each COG category (level 1) was then compared between DGR-encoding genome bins and non-DGR-encoding genome bins for each metagenome using a Kolmogorov–Smirnov test and Cohen’s effect size.

### Phylogenetic analyses

For RT phylogeny, a representative of each RT cluster was selected as the sequence with the highest score when compared to the RT cluster hmm profile, or the longest sequence in case of ties, first among the references if available, then among the new sequences if no reference was present in the RT cluster. An RT tree was then built with IQ-Tree^71^ v1.5.5 using the built-in model selection (optimal model suggested: VT + F + R10), based on an amino acid multiple alignments computed with MAFFT^45^ v7.407 using the einsi mode and automatically trimmed using TrimAl^70^ v1.4.rev15 with the -gappyout option. Sequences from the non-DGR RT reference set (see above) were included in the alignment, with the exception of sequences identified as “unknown” or “unclassified” by Wu et al., as these led to a lower quality alignment and long-branch attraction issues in the resulting tree.

The distribution of DGR features (genome type, taxonomy, target, and biome) across the tree was analyzed by computing unweighted Unifrac distances^72^ between all pairs of values for each features and comparing these with the distance for the same pair of values on 100 randomly shuffled trees. Ancestral state reconstructions were conducted using the R package phytools^73^ v0.6-99 with the options model = “ER” and type = “discrete”, separately for each feature. The increase in phylogenetic diversity associated with this new extended DGR catalog was calculated as the total length of all branches leading to cluster(s) entirely composed of metagenome-derived sequences described in this study, divided by the total length of all branches leading to clusters including at least one of the 366 reference DGR sequences described in ref. ^2^.

A similar pipeline was used to build the trees of target sequences from PC_00003. Briefly, for PC_00003 targets, high-quality target protein sequences (see above) clustered into PC_00003 were gathered and dereplicated at 80% amino acid identify using cd-hit^44^ v4.8.1. A multiple alignment of representative sequences was then generated with MAFFT^45^ v7.407 using the einsi mode, automatically trimmed using TrimAl^70^ v1.4.rev15 with the -gappyout option, and used as input to build a tree with IQ-Tree^71^ v1.5.5 with built-in model selection (optimal model suggested: WAG + F + R10).

### Phylogenetic logistic regression between DGR presence in a genome and biome type

Two trees were built for phylogenetic logistic regression, one for cellular-encoded DGRs, and one for viral-encoded DGRs. For cellular-encoded DGRs, all bacterial genome bins from the GEM dataset^26^ were searched for the presence of the phylogenetic marker RNA Polymerase beta subunit (RpoB) based on IMG annotation to the pfam domain PF04563. For genome bins with multiple hits to this pfam domain, the sequence with the highest score was retained. The corresponding RpoB protein sequences were clustered at 90% identity using cd-hit^44^ v4.8.1 to get a non-redundant set. Next, RpoB sequence representatives from DGR-encoding genome bins (previously identified, see above) were compared with blastp^41^ v2.9.0+ to all RpoB representatives (maximum *e*-value: 1E−03), and the 40 most similar sequences (i.e., best blastp score) from non-DGR-encoding bins were collected. This yielded a dataset of 2169 sequences, including 174 from DGR-encoding bins. These 2169 were used as input in a standard tree building pipeline including multiple alignments with MAFFT v7.407^45^ using the auto mode, automatic trimming using TrimAl v1.4.rev15^70^ with the -gappyout option, and tree built with FastTree^46^ v2 using the LG model. A similar approach was used for viral-encoded DGRs, using the Terminase large subunit (TerL) as a viral marker gene. First, contigs from the IMG/VR v3 database^74^ (i.e., independently identified as viral) were searched for TerL sequences using hmmsearch v3.2.1^40^, using pfam domains PF03354, PF04466, PF03237, and PF05876 (maximum *e*-value: 1E−05). Next, viral contigs encoding both a DGR and a TerL gene were identified, the corresponding TerL sequence extracted, and compared to all TerL sequences from IMG/VR high-quality viral genomes via blastp v2.9.0+^41^ (maximum *e*-value: 1E−03). For each TerL sequence associated in a DGR-encoding genome, the 40 most similar sequences (i.e., best blastp score) from non-DGR-encoding genomes were collected. The final dataset included 5326 sequences, including 883 from DGR-encoding genomes.

Each tree was then used as input in a phylogenetic logistic regression analysis using the phyloglm function from the phylolm R package v2.6.2^75^. To this end, the presence of DGR on individual genomes (represented by individual marker genes) was coded as “1” if present and “0” if absent, and each genome was also associated with the biome (2-level category) of its original metagenome. Phylogenetic regression was performed with 5000 bootstraps, all other parameters default. For results reported in Supplementary Table 1, the alpha parameter is log-transformed and interpreted as suggested in ref. ^76^, i.e., the phylogenetic signal was considered as significant if the minimum value of a parameter across all bootstrap replicates was > −4.

### Estimation of host range for DGR-encoding phages

To estimate phage host range, we formed a comprehensive database of 6,675,007 CRISPR spacers (497,912 arrays) identified from 576,561 prokaryotic reference genomes (38% with a predicted CRISPR array). These reference genomes were compiled by a previous study^26^ and include 52,515 MAGs from the genomes from earth’s microbiomes (GEM) catalog, as well as all publicly available isolate genomes, MAGs, and single-amplified genomes available at the time of that publication. The 576,561 genomes were previously clustered into 45,599 species level operational taxonomic units (OTUs) based on 95% average nucleotide identity and taxonomically annotated using the GTDB-tk toolkit v0.3^68^. CRISPR arrays were identified using a combination of CRT v1.1^77^ and PILER-CR v1.0.6^78^ with default parameters. Redundant CRISPR arrays predicted by both tools were merged based on genomic coordinates.

To determine the host range, CRISPR spacers were matched against 3575 viral genomes containing a DGR (DGR+ viruses) and 19,761 viral genomes lacking a DGR (DGR− viruses). DGR+viruses were identified as described above, and DGR− viruses were extracted from the IMG/VR v3 database^74^ using only metagenomes containing at least one DGR+ virus, such that DGR+ and DGR− viruses were derived from the same set of samples. Only viral genomes estimated to be >90% complete based on CheckV v0.7.0^79^ were included in this analysis. CRISPR sequence matching was performed using blastn from the blast+ package v.2.9.0^41^ (options: -dust = no -word-size = 18), allowing a maximum of one mismatch or gap over the full length of the spacer.

We used the CRISPR spacer matches to estimate the species-level host range for each virus using two diversity metrics: richness and Shannon’s Entropy. To estimate host richness, we counted the number of distinct host species (i.e., OTUs based on 95% ANI) connected to each virus using a random subset of 50 CRISPR spacer matches. This resulted in 822 DGR+ and 1182 DGR− viruses with at least 50 protospacers. We estimated Shannon’s Entropy for the same subset of viruses, using the entropy function in SciPy (scipy.org). Host range (i.e., richness and entropy statistics) was compared between DGR+ and DGR− viruses using the Wilcoxon rank-sum test in R v3.6.1^80^.

### Diversity estimation of DGR target loci

Nucleotide and amino acid diversity evaluation was conducted on the metagenome-derived DGRs with contig length ≥10 kb and median coverage ≥20×. The coverage cutoff was applied to ensure that single nucleotide polymorphisms (SNVs) could be called with enough certainty, while the length cutoff was used to ensure that enough surrounding genes were available to evaluate background microdiversity for the DGR-encoding genome. For this analysis, combined assemblies (i.e., datasets obtained by combining reads from multiple samples), metatranscriptomes, and viral metagenomes were not included. The final set included 6901 DGRs, with representation of all DGR clades (1968 DGRs from DGR_Clade_1, 972 from DGR_Clade_2, 1359 from DGR_Clade_3, 1147 from DGR_Clade_4, 1095 from DGR_Clade_5, and 350 from DGR_Clade_6).

For DGRs found on contigs ≥20 kb, a region of 20 kb around the RT (i.e., up to 10 kb in 5′ and 3′) was extracted and used in these analyses. Reads from the original metagenome were first recruited to the contig (or selected contig subset if the original contig was ≥20 kb) using bwa^81^ v0.7.17-r1188 (default parameters). Reads which matched the reference sequence on at least 50% of their length were then extracted using filterBam (https://github.com/nextgenusfs/augustus/tree/master/auxprogs/filterBam) and realigned against the same reference sequence using bbmap^82^ v38.73 to obtain a global alignment of the read to the reference contig instead of the local/soft-clipped alignment provided by bwa (bbmap options “vslow minid = 0 indelfilter = 2 inslenfilter = 3 dellenfilter = 3”, see Supplementary Note 11). This global alignment was required to accurately estimate SNVs in regions with a high number of mismatches, such as VRs with many different variants in the population. Typically, in these regions, local alignment tools will either trim the mapping to remove these mismatch-containing regions, or “soft-clip” them, i.e., mask them in the resulting sam file, which eventually means that no SNV will be called in these variable regions. Instead, by re-aligning the same reads with a global alignment algorithm, all positions from the read will be considered and SNVs can be identified.

SNVs were called using bcftools^83^ v1.9 “mpileup” and “call” functions (options “-A-Q 15-L 8000-d 8000” for mpileup, “-ploidy 1-A-m” for the call). Only SNVs for which the alternative allele was supported by ≥4 reads or ≥1% of the reads (whichever was smaller) were further considered. These SNVs were then classified as synonymous or non-synonymous based on the available gene prediction, and used to calculate pN/pS for each gene as in Schloissnig et al.^33^. Another set of SNVs was called using FreeBayes^84^ v1.3.1 using the options “-ploidy 1-min-base-quality 15-haplotype-length 0-min-alternate-count 1-min-alternate-fraction 0-pooled-continuous-limit-coverage 8000” and the same cutoff on read representation. The two SNV sets were found to be mostly overlapping (see Supplementary Note 11), and the bcftools SNVs were used in all subsequent analyses.

To evaluate DGR activity, an enrichment of the VR locus in non-synonymous SNVs was calculated as follows. For each DGR, the frequency of non-synonymous SNVs was first calculated as the average number of non-synonymous SNVs per position observed across all genes for the contig (or contig subset). A Poisson law was then used to compare the number of non-synonymous SNVs observed in the VR locus to an expected number of non-synonymous SNVs based on surrounding genes. All cases for which the number of non-synonymous SNVs observed in the VR locus was significantly higher than expected by chance (Poisson law probability <0.05) were considered as observations of DGR activity. For cases where different metagenomes were available from the same sample, only the observation with the highest coverage was selected and used in DGR activity evaluation. While no significant correlation was detected between SNP density and coverage for the sequences selected here (Pearson correlation *p*-value > 0.25), we did notice a higher fraction of DGRs identified as “inactive” in the lower coverage range, which suggests there may be some level of under-detection of DGR activity for these ~20–50× coverage (Supplementary Fig. 12).

To further explore the dynamics of VR loci in nature, 130 datasets were identified as longitudinal sampling that enabled tracking of DGRs across time in the same system (Table S7, including data previously analyzed in refs. ^36,37,85–88^). For these, a similar mapping approach was used as described above, but including all metagenomes from a single subject (for human-associated metagenomes) or location (e.g., geographic coordinates and water layer). The same approach based on non-synonymous SNV frequencies was used to evaluate DGR activity for each individual sample, except that the minimum median coverage was set at 10×. For cases in which multiple metagenomes were available for a single subject/location and time point, the one with the highest coverage was used for each DGR. In addition, nucleotide diversity (Π) was calculated for each VR locus as the average nucleotide diversity observed for each position. To evaluate changes in the VR locus between samples, amino acid variants were called using Anvi’o^31,32,59^ v6.1 based on the same read mappings. This enabled us to accurately evaluate the frequency of individual amino acid residues, including the ones for which multiple positions in the codon were variable.

### Data processing and visualization

Plots and charts were generated in R^80^ v3.6.1 using the ggplot2^89^ v3.2.1 package, while phylogenies were visualized using iToL^90^ v4 (https://itol.embl.de/) and predicted protein structures were visualized with UCSF Chimera^91^ v1.11.2. Several programs used in this study benefited from the GNU parallel tool v20190722^92^. The genome maps from Supplementary Fig. 4 were generated using Easyfig^93^ v2.2.3.

### Reporting summary

Further information on research design is available in the Nature Research Reporting Summary linked to this article.

## Supplementary information

Supplementary Information

Peer Review File

Description of Additional Supplementary Files

Supplementary Data 1

Supplementary Data 2

Supplementary Data 3

Supplementary Data 4

Supplementary Data 5

Supplementary Data 6

Supplementary Data 7

Supplementary Data 8

Supplementary Data 9

Reporting Summary

## Data Availability

All metagenome assemblies are available through IMG (https://img.jgi.doe.gov) using accession numbers listed in Supplementary Data 1. Additional processed results are included as Supplementary Data as follows:- All_RTs.faa: fasta file of amino acid sequences for all RTs used in the analysis (DGR and non-DGR). All_DGR_targets.faa: fasta file of amino acid sequences for all predicted DGR targets. Note that this file includes all sequences predicted by the automatic pipeline to be a DGR target, before any of the manual curation steps mentioned in the Methods section. All_DGR_TR_VR.fna: fasta file of nucleotide sequences for TR/VR pairs. As for the targets, this file includes all sequences predicted by the automatic pipeline, before any of the manual curation steps mentioned in the Methods section and Supplementary Notes. The same files are also available at: https://bitbucket.org/srouxjgi/dgr_scripts/src/master/Companion_datasets/. Other databases used in the study include: Pfam v31—the entire database was used for functional annotation—http://pfam.xfam.org/—NCBI GenBank (sequences downloaded on January 23 2019) —reference DGR sequences were obtained from GenBank, sequence identifiers are listed in Supplementary Data 2—https://www.ncbi.nlm.nih.gov/—Pdb79 v 190918, Pfam v32, and SCOPe70 v1.75, provided as part of the HH-suite package—the full databases were used for functional annotation—http://wwwuser.gwdg.de/∼compbiol/data/hhsuite/databases/hhsuite_dbs/—Gold, i.e., Genome Online Database (accessed on April 19, 2019)—Ecosystem classification was obtained for all samples using the identifiers listed in Supplementary Data 1—https://gold.jgi.doe.gov.

## Code availability

Scripts used in this study are available at https://bitbucket.org/srouxjgi/dgr_scripts/. The version of the scripts used in the manuscript corresponds to the “v1.0” release/tag.
