# Supplementary material for: Ecology and molecular targets of hypermutation in the global microbiome
Source: Nat Commun. 2021 May 24;12:3076. doi: 10.1038/s41467-021-23402-7 (PMC8144416; doi:10.1038/s41467-021-23402-7)
Supplement: Supplementary file 1 — Supplementary Information [file 41467_2021_23402_MOESM1_ESM.pdf]

Ecology and molecular targets of hypermutation in the global microbiome.

Supplementary Information

Simon Roux[1*], Blair G. Paul[2], Sarah C. Bagby[3], Stephen Nayfach[1], Michelle A. Allen[4], Graeme Attwood[5], Ricardo Cavicchioli[4], Ludmila Chistoserdova[6], Robert J. Gruninger[7], Steven J. Hallam[8,9,10,11,12], Maria E. Hernandez[13], Matthias Hess[14], Wen-Tso Liu[15], Tim A. McAllister[7], Michelle A. O'Malley[16], Xuefeng Peng[17], Virginia I. Rich[18], Scott R. Saleska[19], Emiley A. Eloe-Fadrosh[1*]

[1] DOE Joint Genome Institute, Berkeley, CA, USA
[2] Marine Biological Laboratory, Woods Hole, MA, USA
[3] Department of Biology, Case Western Reserve University, Cleveland, OH, USA
[4] The University of New South Wales, Sydney, New South Wales, Australia
[5] AgResearch Limited, Grasslands Research Centre, Palmerston North, New Zealand
[6] University of Washington, Seattle, WA, USA
[7] Lethbridge Research and Development Centre, Agriculture and Agri-Food Canada, Lethbridge, Alberta, Canada
[8] Department of Microbiology & Immunology, University of British Columbia, Vancouver, Canada
[9] Graduate Program in Bioinformatics, University of British Columbia, Genome Sciences Centre, Vancouver, Canada
[10] Genome Science and Technology Program, University of British Columbia, Vancouver, Canada
[11] Life Sciences Institute, University of British Columbia, Vancouver, Canada
[12] ECOSCOPE Training Program, University of British Columbia, Vancouver, Canada
[13] Instituto de Ecología A.C. Red de Manejo Biotechnológico de Recursos. Xalapa, Veracruz, México
[14] University of California Davis, Davis, CA, USA
[15] University of Illinois at Urbana-Champaign, Urbana, IL, USA
[16] Department of Chemical Engineering, University of California Santa Barbara, Santa Barbara, CA, USA
[17] Marine Science Institute, University of California Santa Barbara, Santa Barbara, CA, USA
[18] Ohio State University, Columbus, OH, USA
[19] University of Arizona, Tucson, AZ, USA

[*]Correspondence to: sroux@lbl.gov & eaeloefadrosh@lbl.gov

SUPPLEMENTARY NOTES

1. Iterative detection of DGR in IMG public genomes and metagenomes

Overall, up to 3 rounds of DGR detection were performed for both genomes and metagenomes. The first round of detection was based on known HMM profiles of DGR RTs, while after each round, new profiles were generated from the DGR RT sequences collected, and used as references for the next searches (see Methods). For whole genome shotgun sequences (i.e., “IMG isolates”), the first round of searches identified 2,793 candidate DGR sequences, the second 442 candidates, while the third yielded only one candidate that was identified as a false-positive, hence no further searches were performed. For metagenomes, the first round identified 45,704 candidates, the second 10,964 candidates, while the third round provided seven candidates which were all identified as false-positives. Overall, DGRs were detected across 1,129 genomes and 2,684 metagenomes.

For both genomes and metagenomes, false-positive detections were mostly associated with RTs encoded on eukaryote genomes, especially in regions containing multiple imperfect repeats with seemingly random mismatches and both repeats within predicted CDS, as opposed to the A bias and one intergenic repeat of typical DGRs. When included in a phylogenetic tree based on RT protein sequences, these candidates formed a clade outside of all known RTs, and in particular branched outside of the known DGR clade. Because these sequences are likely not representing genuine DGRs despite the presence of nearby repeats, the choice was made to only retain sequences with a typical DGR mismatch profile (i.e., enriched in A mismatches) or branching within the known DGR clade, while all other candidates were excluded.

While a majority of DGR-encoding contigs could only be affiliated to the phylum or class rank, a total of 4,755 were affiliated up to the genus rank, distributed across 369 bacterial genera and 9 archaeal genera. Even though 48% of these affiliations were to only 3 genera (*Bacteroides*, *Pseudomonas*, and *Prevotella*), both because of a high prevalence of DGRs in these taxa and an over-representation of these sequences in the metagenomes mined, the less common genera revealed new DGR-encoding taxa (Supplementary Data 2). These included notably members of the *Fibrobacter* genus, key actors in the degradation of cellulose compounds in ruminant animals, for which DGRs have not yet been described and explored. This dataset also included additional examples of DGR for ecologically-relevant genera within the phyla *Chlorobi* (e.g. *Pelodictyon*, *Chlorobaculum*, and *Chlorobium*), *Actinobacteria* (e.g. *Bifidobacterium*, *Colinsella*, and *Gardnerella*), and *Nitrospirae* (e.g. Candidatus *Magnetobacterium*), for which only a handful of examples have been reported. Within the Archaea domain, DGRs were associated with various members of the *Euryarchaeota* phylum and DPANN supergroup.

For this taxonomic affiliation, viral-encoded DGRs were associated with the taxon of their host when available, based on the detection of an integrated provirus or matches to known CRISPR spacers (see Methods). However, the viral genomes encoding these DGRs can also be classified in a separate viral taxonomic framework. When affiliated, nearly all DGR-encoding viruses (n=3,218) were classified in the *Caudovirales* order (either affiliated to an existing *Caudovirales* genus or connected to the main *Caudovirales* component in vContact2 network). Notably, while many giant viruses have been recently identified from metagenome assemblies[1], no DGR were detected in these genomes or co-localized with an NCLDV marker gene, suggesting that DGRs are rare or absent from these large eukaryotic viruses despite their ability to exchange genes with bacteriophages. The only exceptions were two sequences (Meta_3300029305_Ga0307249_1003718615 and Meta_3300018430_Ga0187902_100048955) identified as putative inoviruses because of the presence of an inovirus ATPase marker nearby[2]. However, in both cases, the metagenome contig was too short to distinguish whether the DGR was encoded by the inovirus genome or by a neighboring *Caudovirales* prophage, as previously observed[2].

Hence, overall, the available affiliations of DGR-encoding virus contigs suggest that these elements are mostly, and maybe exclusively, encoded by *Caudovirales*.

2. Homogeneity of DGR OTUs and Clusters

DGR OTUs were defined based on a 95% AAI clustering of the RT sequences, and were homogeneous in terms of taxon, biome, and genome type (i.e., viral vs cellular). Specifically, 99%, 91%, and 94% of non-singleton DGR OTUs were associated with a single taxon, biome, and genome type, respectively (Supplementary Fig. 2). A majority (60%) of DGR RT sequences remained as singletons after this 95% AAI clustering, and >88% of DGR RTs were found in OTUs comprising less than 5 sequences, illustrating how large the DGR RT sequence space is.

A similar pattern was observed for DGR clusters (≥50% AAI groups), of which 99%, 85%, and 96% were associated with a consistent taxon, biome, and genome type, respectively (Supplementary Fig. 2). The lower percentage of consistency for the biome feature was due in part to overlap between connected environments, e.g. “Landfill” and “Groundwater”, as well as the detection of DGR clusters with a broad ecological distribution such as Meta_3300009658_Ga0116188_10269693, which includes members from wastewater treatment plants, biogas reactors, saline and freshwater lakes, elephant gut microbiome and moose rumen microbiome (Supplementary Data 4). While an exception, this suggests that at least some DGR clusters are broadly distributed in the environment. Conversely to the OTU clustering, most DGR RTs were found in a cluster comprising 2 or more sequences, and only 52% of DGRs were found in clusters including less than 5 sequences. In addition, the 11 largest clusters alone gathered >36% of all sequences, illustrating how this dataset enabled the detection of the predominant groups of DGRs in the environment. Consistently, the 11 largest clusters all included at least one reference sequence (Supplementary Fig. 1).

3. Definition of major DGR clades

In order to partition the large RT phylogeny into meaningful clades, we sought to leverage the key features of each DGR cluster including genome type, taxon, and biome. We first mapped each parameter to the RT phylogeny rooted on non-DGR RTs and verified that the distribution of each parameter across the tree was statistically structured and not random (non-weighted Unifrac *p*-value <1E-03). Then, we reconstructed ancestral states for each parameter at each node throughout the tree, and identified deep-branching clades with ancestral states predicted with ≥95% confidence as the main groups of DGRs. Taken together, the different features mapped onto the tree suggest the successive emergence of 6 major DGR clades from a single origin (Fig. 1A & B).

Based on these ancestral state reconstructions, the deepest-branching clade of DGRs (DGR clade 1) was found primarily in host-associated microbiomes (>99% confidence) and encoded on viral genomes (>99% confidence) infecting mostly *Bacteroides* and *Firmicutes* hosts. DGR clade 4 showed similar characteristics with a clear association with host-associated microbiomes (>99% confidence) and *Firmicutes* (>99% confidence), and most sequences found in a viral genome. Two large groups of cellular-encoded DGRs (DGR clades 2/3 and DGR clade 5) branched next to DGR clade 4 suggesting two potentially distinct horizontal DGR transfer events from viruses to bacteria/archaea. In the group including clades 2 and 3, the deep-branching nodes are associated with aquatic (>99% confidence) cellular-encoded DGRs (>99% confidence) affiliated to the CPR (Candidate Phyla Radiation) taxon (>96% confidence). However, another type of DGR is nested within these CPR-encoded DGRs, also originally associated with aquatic environments (>99% confidence) but affiliated to other bacteria taxa, especially *Proteobacteria*. The deep-branching CPR-associated DGRs were identified as “DGR Clade 2”, while the latter clade was identified as “DGR Clade 3”. The other group of cellular-encoded DGRs, “DGR clade 5” branched separately from clades 2 and 3 but was also predicted to originally represent

aquatic (>99% confidence) cellular-encoded (>98% confidence) DGRs. A single clade of viral-encoded (>99% confidence) DGRs branched within it however, suggesting a secondary transfer this time from cellular to viral genomes. This latter clade was identified as "DGR Clade 6".

While the diversity of DGR RTs was vastly expanded by metagenome-derived sequences, each of the 6 DGR clades included at least one reference and/or laboratory-characterized DGR sequence[3]. The original DGR identified in Bordetella virus BPP1[4] was affiliated in DGR Clade 6, along with other reference DGRs mostly identified in prophages from *Proteobacteria* and *Firmicutes* genomes, targeting viral structural proteins (Target PC_0002 and PC_00010, Fig. 2, Supplementary Fig. 9). The *Treponema* DGR[5] was affiliated to DGR Clade 5, along with DGR sequences identified in *Spirochaetes*, *Cyanobacteria*, as well as *Archaea* and associated archaeoviruses[6]. Consistent with the majority of DGR Clade 5 sequences, these reference sequences represented the main clade of cellular-encoded DGRs associated with membrane-bound target proteins (Target PC_00001). DGR Clade 4 included another set of DGRs previously identified on prophages, mostly from *Firmicutes* and *Actinobacteria*, and targeting viral structure proteins. The *Legionella* DGR[7] was affiliated in DGR clade 3, along with other references from *Proteobacteria* and *Bacteroidetes* targeting uncharacterized proteins gathered in Target PC_00009. Interestingly, a number of these references correspond to prophage-encoded DGRs, while others, such as the *Legionella* DGR, are encoded on regions of the cellular chromosome that are predicted as "putative mobile genetic element". This suggests that this DGR may have been frequently exchanged between cellular and viral genomes, or may have originated from a provirus and retained on cellular genomes while the rest of the provirus decayed. DGR Clade 2 gathered sequences identified in the CPR group[8] and associated with various uncharacterized targets including Targets PC_00019, PC_00020, PC_00024, PC_00027, PC_00029, and PC_00040. Finally, DGR clade 1 included references from *Bacteroidetes*, *Firmicutes*, and *Actinobacteria* prophages, targeting mostly viral structural proteins (Target PC_00002, Fig. 2, Supplementary Fig. 9).

4. Distinguishing prediction errors from genuine atypical TR-VR sequences

While our detection approach did not rely on the typical mutational bias of DGRs, i.e., mutations associated with A residues, nearly all (99%) DGR clusters displayed a strong bias towards A mismatches (Fig. 1C). The few TR-VR pairs which did not show this pattern could arise either from different mispredictions of the TR-VR regions or from a genuine DGR not constrained by the typical A mutation bias. Upon manual inspection, different scenarios leading to mispredicted TR-VRs were identified:

- Errors in the predicted CDS in the VR region can lead to an incorrect "T" bias, i.e., most of the mismatches correspond to "T" in the TR because the putative target gene is predicted on the opposite strand of the real target gene. This is especially common for VR regions situated near the edge of a contig (i.e., within the first or last ~ 200bp), and we thus discarded such mutation bias profiles based on partial target genes predicted on the edge of a contig.
- In other cases, the CDS prediction overlapping the VR region seemed correct, however it is unlikely to be a genuine DGR target based on functional annotation and a lack of similarity to any other DGR target (known or predicted). In this case, it is most likely that the TR-VR pair was wrongly identified, and the associated mutations biases were also excluded.
- For TR-VR with a plausible target gene and not on the edge of a contig, several cases were identified for which the blast hit used to define the TR-VR region likely extended past these regions. While the TR-VR initially identified did not display an A bias, an internal subset of the alignment could be identified upon manual inspection with (near-)exclusively A mismatches.

Most likely in these cases, near-identical regions next to the TR-VR led to this misprediction, and the bias was corrected to reflect the one of the "inner" TR-VR.

- Finally, some TR-VR pairs were associated with a plausible target gene and did not include any subset with an A bias. No other mutation bias was apparent in any of these TR-VR pairs, i.e., the mismatches observed did not show any enrichment in a specific nucleotide either in the TR or the VR sequence. These could represent either TR-VR sequences that accumulated mutations beyond the ones introduced by the DGR RT, or DGR RTs for which the incorporation of random nucleotides is not strictly associated with A residues. The small numbers of such "atypical" DGRs and their sparse distribution across the tree indicates however that the tendency of DGR RTs to incorporate random nucleotide specifically at template A-residues is both ancestral and conserved, thus most likely associated with biochemcial and/or structural constraints of the RT enzyme itself[9].

5. Interpretation of phylogenetic logistic regression between DGR distribution and biomes

We used a phylogenetic logistic regression on two trees as described in ref. [10], one for cellular-encoded DGRs and one for viral-encoded DGRs, to further disentangle phylogenetic and ecological signal in DGR distribution. While both trees showed significant phylogenetic and ecological signals (Supplementary Data 1), there were substantial differences in the results obtained for cellular- and viral-encoded DGRs. For the viral tree, all significant correlations with a biome type ("Engineered:Landfill", "Host-associated:Human-fecal", "Host-associated:NonHuman-fecal", "Host-associated:Other") were positive correlations, i.e., highlighting environments where viruses tend to encode more DGRs than expected. Two additional biome types show positive correlation which, although not considered significant here, still had *p*-values of 0.006 and 0.003 ("Aquatic:Saline-lakes" and "Engineered:Anaerobic-incubation"). These environments are consistent with the ones highlighted in Fig. 1E as including a high average number of DGRs per genome, and the positive correlations suggest that a set of diverse viruses encode DGRs in these environments. The diversity of these DGR-encoding viruses may be due to a relatively high frequency of horizontal gene transfer in viral genomes[11] and/or to viral-encoded DGRs providing a broad fitness advantage in these environments regardless of the virus' evolutionary history or taxonomy. Notably, this signal would also be consistent with DGR fitness advantage being associated with specific host characteristics (e.g. high host population diversity), since individual bacterial populations are typically infected by a diverse set of phages[12].

In contrast, the only significant correlation in the cellular tree was a negative correlation for the "Aquatic:Other" biome, while all other environments displayed correlation coefficients not statistically different from 0 (Supplementary Data 1). It is notable that this result was obtained while clades of cellular-encoded DGRs (DGR clade 2 and 5) primarily include sequences from only two types of environment ("Aquatic:Freshwater" and "Aquatic:Groundwater", Fig. 1C). We interpret this result as reflecting a situation where DGR-encoding microbes in these two environments tend to be concentrated in a few specific monophyletic clades, while many other microbes from the same environments do not encode DGR. The narrow phylogenetic distribution of cellular DGRs in these environments may reflect: (i) a limited opportunity for horizontal transfer and/or acquisition of DGR for many taxa, for instance if some genomic background are not able to support DGR mutagenic retrohoming, and/or (ii) a limited set of lifestyles and/or metabolisms in which cellular-encoded DGRs provide a selective advantage.

6. DGR targets: *de novo* clustering process, results, and benchmarking

To avoid over-estimating the diversity of target proteins, we applied the *de novo* protein clustering pipeline to "high-quality" (HQ) targets only, i.e., predicted target genes longer than 300 nucleotides and not within 50bp of the edge of the contig. This selection was designed to limit the inclusion of incorrect targets due to partial and/or mispredicted CDS from metagenome contigs. Dereplication (99% AAI) of these high-quality targets led to a dataset of 15,559 non-redundant targets used as input in the two-step clustering process (see Methods). This resulted in 151 protein clusters (PCs) with 2 or more members, designated here as "PC_00001" to "PC_00151". Most (92.18%) high-quality targets clustered in 1 of the 24 largest PCs, which were all associated with plausible functional annotation for DGR targets, and thus further considered as likely genuine DGR targets. Other HQ target sequences found in smaller PCs and/or singletons could be either genuinely rare types of targets or cases for which the target cds or TR-VR regions are misidentified. Because these sequences are mostly originating from short contigs for which genes and DGR features prediction are challenging, we opted to consider these targets as "Rare" and not analyze them further. Non-HQ target sequences were then mapped to HMM profiles derived from these clusters and affiliated to individual PCs if a significant sequence similarity was detected (see Methods).

While the two-step clustering used to establish target PCs enables the identification of remote similarity, which is often required when analyzing viral sequences[13], it may be seen as risking "over-clustering" sequences, i.e., artificially gathering most input sequences in only a handful of PCs. To verify that this was not the case for either viral or cellular proteins, we applied the same two-step clustering pipeline to two "benchmark" datasets: (i) 24,753 random proteins from *Caudovirales* genomes in NCBI Viral RefSeq R201[14], and (ii) 24,987 random proteins from DGR-encoding bacterial/archaeal genomes (Supplementary Data 5). Two clusterings were run for each set of protein sequences, one including the full set, and one including only a random subset of 15,599 sequences (i.e., the same number as HQ target sequences used in our analysis, see above).

In all cases, input sequences were grouped into a much higher number of PCs than the HQ DGR targets, and none of the test clusterings yielded the same pattern of most sequences gathered in a handful of PCs, as observed for HQ DGR targets. Specifically, while >92% of proteins were gathered in only 24 PCs for HQ DGR targets, the 20 largest PCs never included more than 10.2% (for microbial genomes) or 5.6% (for *Caudovirales*) of the input sequences in the benchmarks (Supplementary Fig. 6). Accordingly, Shannon's Entropy calculated from the number of sequences across all PCs was much higher for benchmark clustering (6.69 for microbial genomes, and 7.10 for *Caudovirales* proteins for subsampled benchmarks) than for the DGR targets (4.12), again indicating that the distribution of PC size was much more uneven in the case of DGR targets, with a few PCs encompassing a disproportionate amount of input sequences. Finally, we manually inspected the annotation of PC members for these two benchmark datasets, and verified that these PCs only gathered sequences with consistent functional annotation (Supplementary Data 5). Hence the observation of DGR targets clustering in a handful of PCs is not a methodological artifact but reflects a genuinely low sequence and functional diversity of DGR targets.

7. Examination of putative Ig-like VR domains

Wu et al.[3] previously reported two 'categories" of VRs which were predicted to adopt an Immunoglobulin-like (Ig-like) fold (Ig1 and Ig2). These included respectively 36 and 9 non-redundant targets, with 3 types of domain organization: 25 displayed a C-terminal VR within a short (≤250 aa) protein, 4 included a C-terminal VR with a long (>250 aa) uncharacterized N-terminal part, and 17 had a N-terminal VR followed by a long (>250aa) uncharacterized C-terminal region[3]. As typical for DGR targets, despite the similarities observed between the VR regions, most of these sequences could not be

annotated. Wu et al. however noted that a structure prediction with Phyre 2 suggested that some of these VRs (3 Ig1 and 5 Ig2) may overlap with an Ig-like domain.

In our analysis, Ig1 and Ig2 targets were found in two different PCs, PC_00003 (24 Ig1, 8 Ig2) and PC_00008 (12 Ig1), with one exception (one Ig2 target was clustered in PC_00062, considered a “rare” target in our analysis). Both PC_00003 and PC_00008 are clearly associated with viral-encoded DGRs, which is consistent with previous analysis of Ig1 and Ig2 VRs[3]. Both PCs are also predicted to include a majority of structural (likely tail) proteins (Fig. 2A). However, with PC_00003 and PC_00008 including 1,770 and 416 non-redundant high-quality sequences respectively, this extended dataset provided a unique opportunity to further understand the putative link between VRs and Ig-like domains.

When these targets were annotated using hhblits, members of PC_00003 and PC_00008 did not display any conserved domain overlapping the VR region (Supplementary Data 5). Both members of PC_00003 and PC_00008 had hits to functional domains related to carbohydrate binding outside of the VR regions however, including several with Ig-like domains (Supplementary Data 5). Several types of phage tail fiber proteins have been previously shown to harbor similar Ig-like fold domains[15], which is consistent with expected function of DGR targets. To further confirm that Ig-like domains were commonly found on tail proteins, we annotated 250,209 non-redundant protein sequences from *Caudovirales* genomes in NCBI Viral RefSeq R201[14] using the same pipeline as the one applied to the DGR targets. We identified 1,035 proteins matching an Ig-like domain, including 80.4% annotated as tail- or capsid-related (excluding hypothetical proteins, Supplementary Data 5). This confirmed that proteins containing Ig-like domains are not uncommon in *Caudovirales*, and are for the overwhelming majority structural proteins, most frequently tail fibers.

Ig-like-containing tail fibers have been shown to vary in length, especially through the addition and removal of one or several Ig-like domains in the C-terminal region of the protein[15]. We confirmed this was the case here as well by building a phylogeny of target sequences from PC_00003 and mapping the domain organization of these targets to the tree, two large clades of sequences longer than average and typically including one or several Ig-like domains immediately downstream to the conserved VR domain can be observed (Supplementary Fig. 8 & 9). This would be consistent with the original target displaying a typical C-terminal VR region, and progressively increasing in length through the downstream addition of additional carbohydrate-binding domains, including Ig-like domains, that would not be targeted by DGR retrohoming but located immediately next to the VR (Supplementary Fig. 9).

We further wondered whether these Ig-like domains immediately next to VR regions could lead to structure predictions including an erroneous overlap between VR and Ig-like domains. When we repeated the structural prediction of Ig1 and Ig2 proteins using Phyre2, we observed that all cases for which an Ig-like fold was predicted as overlapping a VR region corresponded to sequences for which multiple Ig-like domains were identified (both by hhblits and Phyre2) downstream from the VR, and found in one of the two clades of sequences longer than average (Supplementary Fig. 9). The structures obtained were also of relatively low quality (as also noted by Wu et al.[3]), and based on templates often consisting of multiple successive Ig-like domains. Conversely, for all Ig1 and Ig2 sequences with a C-terminal VR or with a VR without nearby Ig-like domain, no prediction of an Ig-like fold was obtained. These Ig1/Ig2 sequences are thus most likely phage tail fibers with a non-Ig-like conserved VR domain for which no characterized fold can be identified, but erroneously predicted as an Ig-like fold due to nearby Ig-like domains.

8. DGR targets: Functional annotation of non-VR regions

To evaluate the expansion of DGR target space obtained in our expanded DGR catalog compared to previous studies, we compared the functional annotation obtained on the 24 largest target PCs with the functions of DGR targets previously reported[3]. In addition to conserved domains already detected on reference sequences, 23 domains were newly identified on >5 predicted target sequences. These included conserved domains within S-Layer-containing proteins (PDB 4QVS_A), PEGA domains (Pfam PF08308), putative bacterial lipoproteins (Pfam PF05643), putative glutamyl endopeptidases (PDB 1WCZ_A), serine proteases (PDB 3STI_A and 6BQM_A), and other types of viral structural proteins (PDB 4V96_AG, Pfam PF03906). Overall these confirm that DGR targets are extremely diverse, but are mainly associated with carbohydrate-binding proteins embedded in virions and cell membranes. Notably, among viral-encoded DGR targets, several members of PC_00012 included an atypical eukaryotic-like serine/threonine kinase (CotH-like domain), previously linked to extracellular phosphorylation of membrane proteins[16,17]. While these kinase domains are typically located distantly from the VR, structural predictions suggested both regions may be in close contact, perhaps directly interacting with each other, once the protein is folded (Supplementary Fig. 9). This suggests a potential role beyond host recognition for some of these viral-encoded DGRs, possibly in host membrane modification upon attachment.

For a comprehensive annotation of phage structural proteins, we relied on two approaches more sensitive than sequence similarity: DeepCapTail[18] and PhANNs[19]. PhANNs has been extensively tested and benchmarked[19], and we leveraged these tests to select a threshold of score ≥0.2, which maximizes recall while maintaining a precision and F1-score >0.85. For DeepCapTail, we performed a sensitivity analysis by annotating a set of 250,209 non-redundant set of *Caudovirales* proteins from NCBI RefSeq (Supplementary Data 5). We observed that, even at a conservative threshold of score ≥0.9, a relatively high percentage of sequences (36%) were predicted as likely tail proteins. These included nearly all sequences annotated as “tail”, “tail fiber”, or “tail structure”, in addition to a number of hypothetical proteins and sequences annotated as other functions, which we interpreted as some extent of “over-prediction” (i.e., Type I error) from DeepCapTail. The impact of this marginal over-prediction could be mitigated when DeepCapTail results were considered at the PC level. Specifically, when we clustered the non-redundant RefSeq *Caudovirales* proteins using our two-step clustering pipeline and analyzed DeepCapTail predictions for the 269 PCs with ≥100 members, only 74 PCs included ≥60% of members predicted as capsid or tail proteins by DeepCapTail, and nearly all of these were annotated in RefSeq as structural or virion-related proteins (Supplementary Data 5). Eventually, we combined both predictions for annotating phage-encoded Target PCs as follows (Supplementary Data 5). First, 7 PCs were considered as “tail proteins” because they had ≥60% of their members predicted as a tail structure protein by either or both tools. Specifically, 6 PCs included 54-98% of their members predicted as tail structure proteins across both tools, while 1 PC (PC_00023) included 61% of members predicted as tail structure proteins by DeepCapTail and 39% by PhANNs (Supplementary Data 5). The two other PCs (PC_00009 and PC_00007) were considered as non-structural/unknown, since in both cases PhANNs predicted only a small minority of the sequences as structural (15.8% and 1.6%, respectively, Supplementary Data 5). Since our knowledge of viral structural proteins is still partial[19], it is nevertheless possible that these target PCs currently lacking a functional annotation represent so-far-uncharacterized structural proteins.

Finally, since most of the cellular-encoded DGR targets were predicted to be associated with the cell membrane, we further evaluated targets from bacterial taxa with fundamentally distinct membrane architectures, i.e., monoderm (~ gram-positive) and diderm (~ gram-negative) taxa. We reasoned that, if cellular-encoded DGR targets were indeed membrane-anchored, taxa with different membrane types should encode distinct target PCs. Accordingly, cellular-encoded target PCs partitioned near-perfectly with membrane types (Supplementary Fig. 9). For the 6 target PCs annotated as membrane bound, 5

included ≥95% diderm members, while the sixth (PC_00041) was associated with 100% monoderm members. While a broad range of mechanisms exist to anchor proteins to monoderm cell surface, PC_00041 was annotated as a membrane-bound PC based on the detection of N-terminal transmembrane helix(ces) in >80% of members, suggesting these targets are likely anchored to the cytoplasmic membrane rather than to the cell wall as would be e.g. lipoproteins or LPXTG-like proteins. Meanwhile, target PCs not annotated as membrane-bound were associated with atypical bacteria from the candidate phyla radiation (CPR), or with archaea. This is consistent with previous studies suggesting these two types of organisms include DGRs with atypical target proteins[6,8]. The cellular localization and function of these target proteins remains undetermined at this point, since prediction of membrane localization for these taxa is known to be challenging.

9. Comparison of potential host range between DGR-encoding and non-DGR-encoding viruses

Some viral-encoded DGRs are known to play a role in phage's adaptation to host tropism switching based on experimental studies in *Bordetella* bacteriophages[4]. In this instance, DGR-induced hyper-diversification is used by the phage to cope with the highly dynamic cell surface of its host. Beyond adaptations to change in a single host however, diversification of host recognize proteins could also enable some DGR-encoding phages to attach to a broad diversity of host cells, and possibly expand their host range compared to their non-DGR-encoding counterparts. To test this hypothesis, we connected viruses encoding DGRs (DGR+ viruses) to prokaryotic host genomes using a comprehensive database of 6.7 million CRISPR spacers derived from 576,561 prokaryotic reference genomes, and estimated host range by counting the number of distinct host species connected to each virus. As a control we performed the same procedure for viruses lacking DGRs (DGR- viruses) that were identified from the same metagenomes. To avoid sampling issues, we calculated host range per virus using exactly 50 protospacers, and discarded viruses with fewer than 50 matched CRISPR spacers.

On average, DGR+ viruses were connected to 1.8x as many host species as DGR- viruses (averages = 9.53 and 5.33, respectively, Wilcoxon rank-sum test p-value = 5.3×10^{-96}). A similar pattern was observed using Shannon's Entropy as the measure of host diversity (Supplementary Fig. 10). One potential confounding variable is the number of spacers derived from each host species in the database, since a large pool of spacers from an individual species could result in the appearance of narrow host range. However, to the contrary, DGR- viruses were associated to hosts with smaller spacer pools, ruling out this possibility (Supplementary Fig. 10).

Critically however, connections based on CRISPR spacer matches between a phage and a host species does not mean that this phage is able to successfully infect members of this species. In fact, the integration of a new CRISPR spacer in a host genome is a sign that the corresponding phage successfully entered into this host cell, but then saw its infection aborted by the host cell defense system. Hence, the higher number and diversity of CRISPR connections for DGR-encoding phages should not be interpreted as necessarily a broader host range for these phages, but rather as the indication that, on average, diversification of host recognition proteins does give these DGR-encoding phages the opportunity to attach to and attempt infecting a broader diversity of host cells than non-DGR-encoding ones.

10. Analysis of DGR-encoding metagenome bins

Two analyses were conducted based on DGR identified in IMG genomes bins (see Methods). First, genome bins were used to evaluate whether DGR-encoding viruses infected dominant and/or rare genomes in human gut samples. A total of 124 human gut metagenomes were selected which included at least 10 MQ/HQ bins, at least 1 DGR-encoding bin, and for which coverage information was available. While these metagenomes included 10 to 37 bins, DGR-encoding bins were frequently

among the most abundant genomes observed. Specifically, DGRs were identified in one of the 3 most abundant bins for 68 of these 124 metagenomes (55%). While this pattern may be potentially biased by the fact that genome bins with higher coverage may have a higher completeness than bins with lower coverage, importantly, it was not observed across other biomes. Specifically, when evaluating the number of metagenomes for which a DGR was identified in one of the 3 most abundant bins, these only represented 17 of the 66 qualified metagenomes (26%) from other host-associated samples (i.e., non-human gut), 44 of the 139 qualified metagenomes (32%) from aquatic samples, and 17 of the 67 qualified metagenomes (25%) from engineered samples, compared to 55% for human gut samples. Hence, this pattern is likely not associated with a systematic genome binning bias, and DGRs seem to be specifically associated with abundant members of the community in human gut microbiomes.

Next, we used genome bins to evaluate differences in gene content between DGR-encoding and non-DGR-encoding genomes for Clade 5 DGRs, which are primarily identified in aquatic biomes. Compared to MQ/HQ genome bins from the same metagenomes that do not encode a DGR, genome bins encoding a Clade 5 DGRs displayed a significant enrichment in key COG categories associated with copiotrophic and/or particle-associated lifestyle. Specifically, genome bins which included a Clade 5 DGR showed a higher percentage of genes assigned to COG category N “Cell motility”, T “Signal transduction mechanisms”, and V “Defense mechanisms” compared to other bins from the same metagenomes (ks-test *p*-value $\leq$1E-5, Cohen’s effect size $\geq$0.2), all categories previously highlighted as enriched in copiotrophs[20]. Importantly, the same pattern, i.e., significant enrichment in COG categories N, T, and V, was observed for bins including the other main clade of cellular-encoded DGRs, DGR clade 3, but not for any other clade. This indicates that these patterns are not a systematic bias of genome binning, but instead reflect key features in terms of gene content of micro-organisms encoding DGRs of clade 3 and 5. Given that the target genes of DGR clade 5 are typically membrane-bound, it is tempting to speculate that at least some of the DGRs in these clades drive hyper-diversification of surface protein directly involved in particle binding, and would thus broaden the range of particles and/or other microbial cells to which a microbe could bind.

11. Evaluating DGR activity from read mapping

Because of the high number of SNVs concentrated in a short region, mapping pipelines for VR sequences must use specific parameters to allow more mismatches and avoid under-recruiting short reads to the hypervariable reference. To achieve this, we used here a two-step mapping process. First, all reads were mapped using bwa to recruit all reads matching, even partially, to the reference sequence (i.e., based on local alignment). Then, reads which were locally aligned on at least 50% of their length were mapped against the same reference using bbmap with parameters tuned toward optimization of the read global alignment and tolerating mismatches (see Methods). We verified whether most reads from VR regions were likely recovered by comparing the read depth of VR regions to the one of surrounding genes from the same contig (Supplementary Fig. 11). Since some metagenomes will display variable read coverage along a single contig, e.g. due to PCR amplification of the library[21], we established a 95% confidence interval for coverage along each contig based on the average coverage of non-target genes minus 2 standard deviations, and considered as “low coverage” cases in which the VR coverage was below this cutoff. Pragmatically, we considered VR region with a coverage below the average minus 2 standard deviations cutoff as unexpectedly low, and likely reflecting an incomplete recruitment of VR reads. Overall, >91% of VR regions displayed a coverage above the 95% confidence interval lower bound, confirming that most reads coming from these VR regions had been recovered.

To further verify that SNVs could be robustly called in VR regions, we compared the number of SNVs detected by two different tools: bcftools mpileup/call and freebayes (see Methods). Overall, both SNV calling approaches produced very similar result (Supplementary Fig. 11), resulting in a Pearson

correlation coefficient of 0.873 (95% confidence interval: 0.868-0.879, *p*-value <2.2e-16) between SNV densities for individual VR regions. We thus proceeded using only one of these SNV sets, and opted to use the more conservative one given the parameters used here, i.e., bcftools (Supplementary Fig. 11).

In order to measure the selective constraints exerted on individual genes and/or VR regions, we first relied on the known pN/pS metric, calculated as in Schloissnig et al.[22]. For non-target genes, this pN/pS ratio was on average 0.16 (95th percentile=0.60), as expected for microbial genes evolving under long-term purifying selection. By contrast, pN/pS ratio average was 2.83 for target genes. Notably, pN/pS calculations were sometimes impossible to calculate because of an absence of synonymous SNVs. While in the case of non-target genes, the absence of synonymous SNVs was mostly (>90% of the time) associated with an absence of non-synonymous SNVs, this was not the case for VR regions, of which 51% displayed ≥1 non-synonymous SNVs but 0 synonymous SNVs. In order to include these sequences in the activity estimation, we opted to use an "enrichment in non-synonymous SNVs" statistics comparing the density of non-synonymous SNVs in a VR region to the one in surrounding non-target genes. The two approaches were largely congruent, as for cases in which both could be calculated, the 296 sequences without an enrichment in non-synonymous SNVs all had low pN/pS (median=0.39), while the 2,417 sequence with an enrichment in non-synonymous SNVs had a high pN/pS (median=2.48).

Finally, we searched for time-series datasets that could provide insights into the population diversity of DGR loci through time. We first identified time series among our metagenome set, and linked each sample to its "subject/location", i.e., individual patient for human cohorts, individual bioreactor for laboratory incubations, individual water body and/or depth layer for lakes (Supplementary Data 6). Individual water layers were used as subject/location when they represented distinct ecological conditions, or were grouped as a single subject/location otherwise to avoid duplicate observations. Candidate DGRs for time series analysis were identified based on DGR OTUs which included members assembled from multiple datasets of a single subject/location. For these, reads from all datasets associated with the subject/location were mapped to the same DGR OTU representative sequence, and the longitudinal analysis was conducted if the median coverage of this sequence was ≥10x in ≥2 time points. Overall, 563 DGR OTUs were analyzed this way across 130 time series, and covered all DGR clades (the lowest number of DGR OTUs was for DGR clade 2, with 47 OTUs).

12. Definition of activity categories for time series

The activity of DGR analyzed as part of a time series were evaluated based on single amino acid variants[23] called using Anvi'o (see Methods), i.e., for each position of interest and each sample, a vector of frequency of amino acid alleles was determined by Anvi'o based on read mapping. For each TR-VR pair, all amino acid residues in the VR for which at least one of the position in the codon corresponded to an A in the TR were evaluated, along with 10 randomly chosen positions upstream in the target protein sequence which were used as control.

Three complementary metrics were computed from these amino acid alleles frequency vectors. First, the entropy calculated by Anvi'o was used as a measure of the populations diversity at a given position in a given sample. Based on the overall distribution of entropy values across VR and control positions, we established three categories of positions: low entropy for values ≤0.25, medium entropy for values >0.25 and ≤0.5, and high entropy for values >0.5. Next, we calculated for each position a cosine similarity between the allele frequency vector of a sample and the allele frequency vector of the previous sample in the time series. Again, based on the overall distribution of cosine similarities across consecutive time points for VR and control positions, we defined cosine similarity values ≥0.9 as "high similarity", values ≥0.75 and <0.9 as "medium similarity", and values <0.75 as "low similarity".

Finally, we calculated the number of changes in the dominant (i.e., majority) allele throughout the time series for each position.

Four categories of DGR activity were then defined based on a combination of these 3 metrics. First, if the entropy of position was always high, i.e., >0.5 for all time points, the position was considered as "constant diversity", i.e., it is likely that the corresponding DGR is active enough to counteract any purifying selection. If instead a position included in the same time series included both samples with low (i.e., ≤0.25) and high (i.e., >0.5) entropy, it was considered as "alternating", i.e., these changes were interpreted as a series of DGR-driven diversification events followed by diversity reduction through purifying selection and/or drift. Alternatively, if at least one dominant allele change was observed during the time series with a low cosine similarity (i.e., <0.75), the corresponding DGR was also considered as "alternating". In this case, we interpreted the change in dominant allele associated with a high distance between allele frequency vectors as evidences suggesting some unsampled DGR diversification event between the time points. The cutoff on distance between allele frequency vectors enabled us to distinguish cases with genuine changes in the amino acid composition of a position, from cases with multiple co-dominant alleles of nearly equal proportion, for which a change in dominant allele could be observed by chance without the need for any diversification or selection event. Positions not considered as "constant diversity" or "alternating" were then classified as "constant selection" if the minimum entropy was at least medium (i.e., >0.25). These are cases for which few to no change in the dominant allele are observed, however all samples show significant population diversity suggesting a continuous DGR-driven diversity likely controlled by purifying selection. Finally, other positions with either all samples with entropy ≤0.25 (low entropy) or all similarities between samples ≥0.9 (high similarity) were considered as "inactive". We chose to interpret these as sign that the corresponding DGR was not active, although similar allele frequency profiles could be obtained from active DGR associated with very strong purifying selection if the fitness of different variants was constant across the time series.

Overall, >97% of the "control" positions (i.e., positions from the target gene but not in the VR region) were classified as "inactive", as would be expected for positions under strong purifying selection. In contrast, only 43% of the VR positions were classified as "inactive", despite the fact that the VR regions were determined automatically and likely include non-VR positions in 5' and 3' of the actual TR-VR repeat. The other VR positions distributed between "constant diversity" (35%), "alternating" (15%), and "constant selection" (7%). This is consistent with the high rate of non-synonymous SNV identified from individual metagenome mapping (Fig. 3A & B), and confirms that the population diversity observed at VR locus is fundamentally different from the one observed at other positions even in the same target gene and the same samples.

13. Estimation of the contribution of DGRs to overall amino acid changes in viral genomes

To conservatively estimate the contribution of DGRs to overall amino acid turnover in viral genomes, we first selected DGRs from clades 1, 4, and 6 for which longitudinal data was available, and excluded data from laboratory incubations (Supplementary Data 6). For each type of dataset ("Temperate_Lakes", "Antarctic_Lakes", "Human_microbiome", "Human_microbiome_perturbed"), the total number of changes in dominant allele observed on VR and control positions (see above) was tallied and divided by the total number of observation for each group to obtain a "frequency of change" for each group. Based on an average genome size of ~50kb and coding density of ~90% for *Caudovirales* (based on genomes in NCBI Viral RefSeq v93), we estimated an average number of position per genome of 15,000 (45,000 nucleotide positions in protein-coding gene leading to 15,000 codons/amino acid residues). For VR positions, we used the average number of VR positions predicted by DGR, i.e., 17. For each dataset, the average frequency of change for each group (background and

VR) was thus multiplied by the estimated number of positions for each group in an average genome (15,000 and 17) to obtain estimates of total number of changes for each group. The ratio between the number of changes in VR and the total number of changes was then used as an estimate of the contribution of DGRs to amino acid turnover in an average DGR-encoding viral genome.

The estimated proportions of amino acid changes associated with DGRs were 6.14% in “Human_microbiome”, 7.32% in “Human_microbiome_perturbed”, 9.67% in “Temperate_Lakes”, and 16.35% in “Antarctic_Lakes”. Importantly, we consider these estimates as conservative because all positions randomly selected as ‘control’ were taken from outside the predicted VR but within the same DGR target gene. These genes are likely to experience more frequent changes even outside of the VR region than other housekeeping genes because most of them are directly involved into virus-host interactions. Hence, these estimated proportion of DGR-driven amino acid changes should be seen as lower boundaries of the actual value.

SUPPLEMENTARY FIGURES

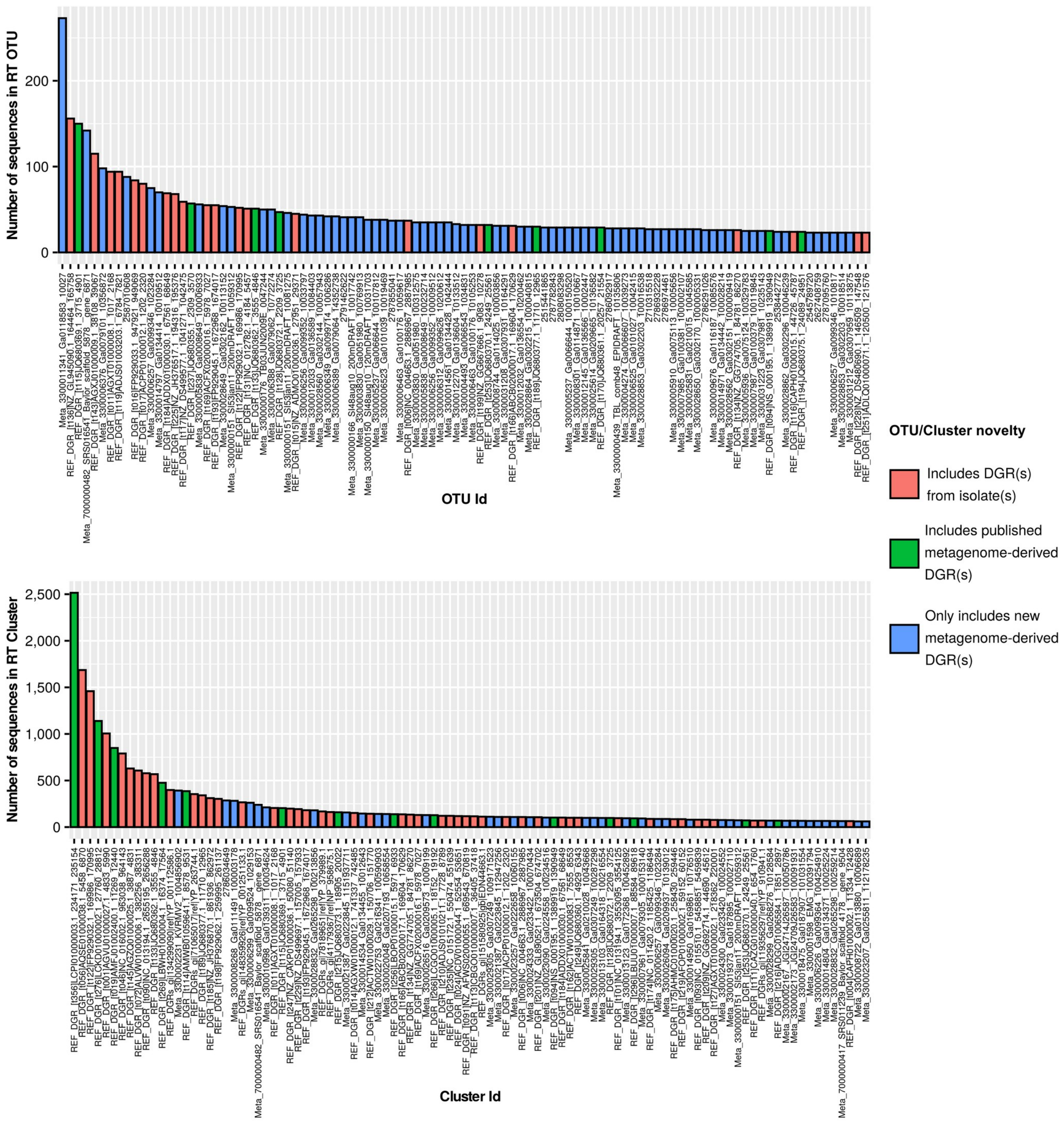

Supplementary Figure 1. **Size distribution of the 100 largest RT OTUs (top panel) and RT Clusters (bottom panel).** The bars are colored according to the presence/absence of reference sequences in the corresponding OTU/Cluster.

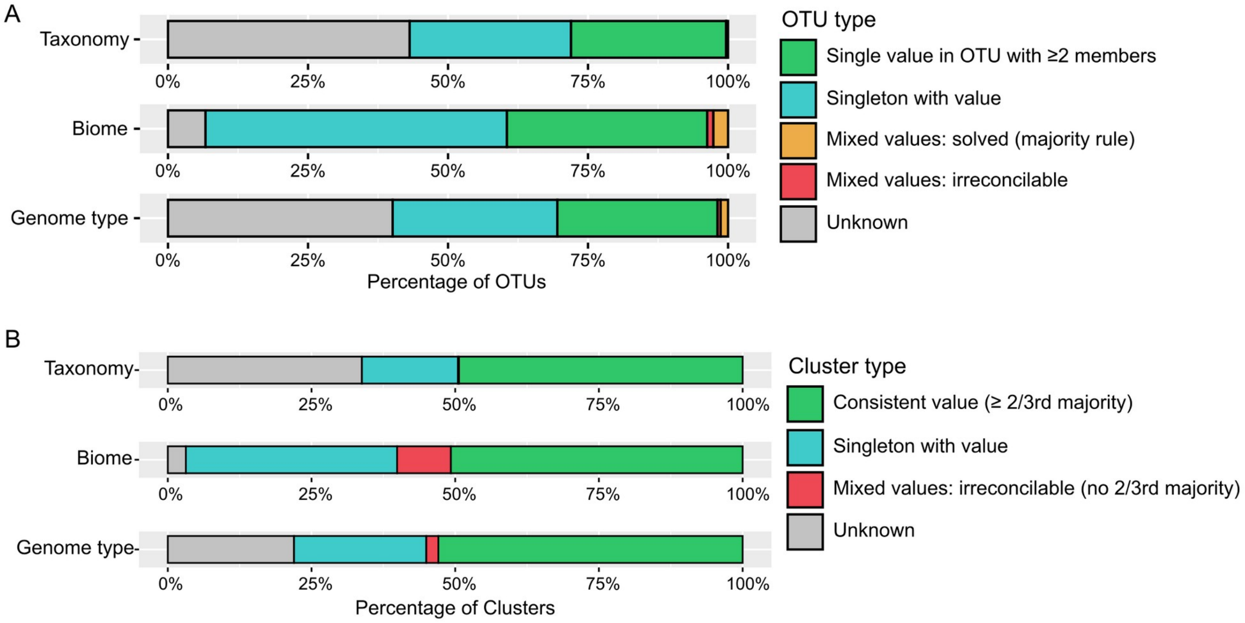

Supplementary Figure 2. **Characteristics of DGR RT OTUs and Clusters.** Each bar chart indicates the consistency of one feature (taxonomic classification, biome, or genome type) across members of a DGR RT OTU (A) or DGR RT Cluster (B). For OTUs with ≥2 members (i.e., non-singletons) with inconsistent values for a feature, a majority rule was applied: if a majority of an OTU members had the same value, this value was used for the OTU ("Mixed values: solved"). In case of tie (i.e., equal number of members associated to different features), the OTU feature was considered as unknown ("Mixed values: irreconcilable"). For Clusters, a similar approach was used with a 2/3rd majority rule. All Clusters for which ≥2/3rd of the members had the same value were considered as "Consistent value" and the value was assigned to the cluster. Cases in which the majority value in the cluster was associated with <2/3rd of the members were considered as "Mixed values: irreconcilable".

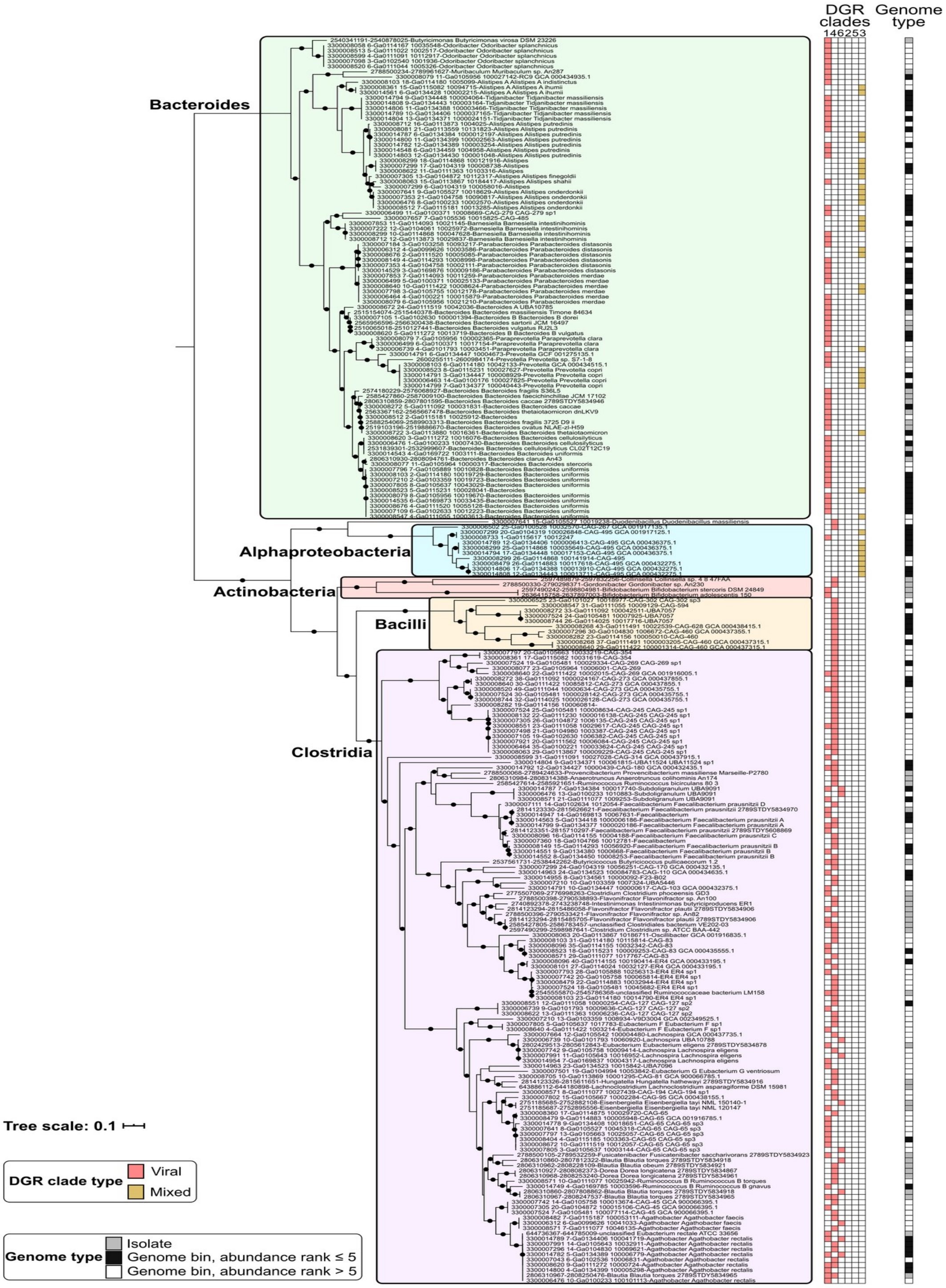

Supplementary Figure 3. **Phylogeny of isolate and metagenome-binned genomes encoding one or more DGRs and associated with human gut samples.** Nodes with support <50 were collapsed, and nodes with support ≥80 are noted with a black circle. For each genome, the different clades of DGRs detected in the genome is indicated next to the tree as a colored heatmap. The genome relative abundance is then indicated next to the heatmap: isolate genomes are highlighted with grey squares, genome bins ranked as one of the 5 most abundant genomes within a metagenome are highlighted with black squares.

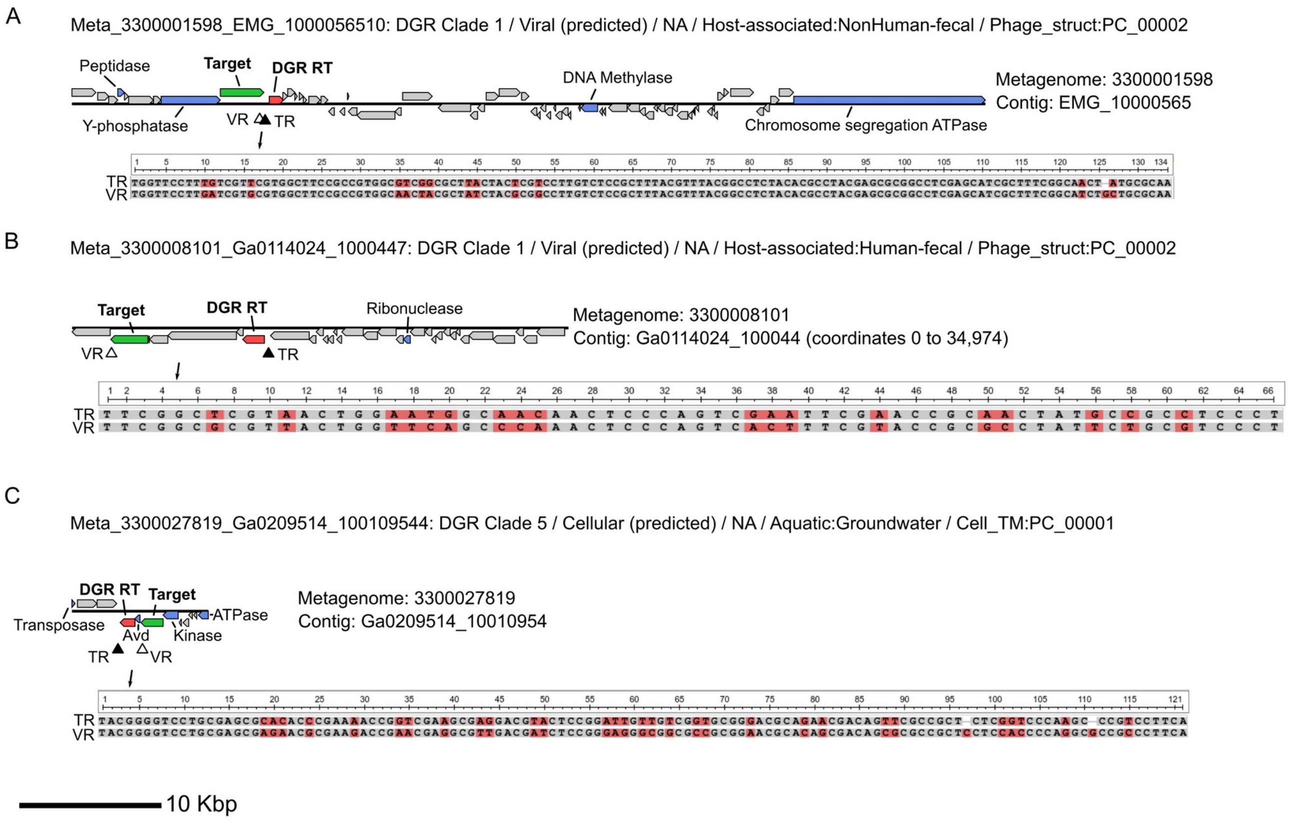

Supplementary Figure 4. **Examples of predicted DGRs with atypical (non-A) mutation bias.** For each DGR, the clade, genome type, taxonomic classification, biome, and primary target affiliation are indicated when available. The genome maps are colored based on each predicted CDS functional annotation: the DGR reverse-transcriptase in red, target gene in green, other genes in blue, and “hypothetical protein” in grey.

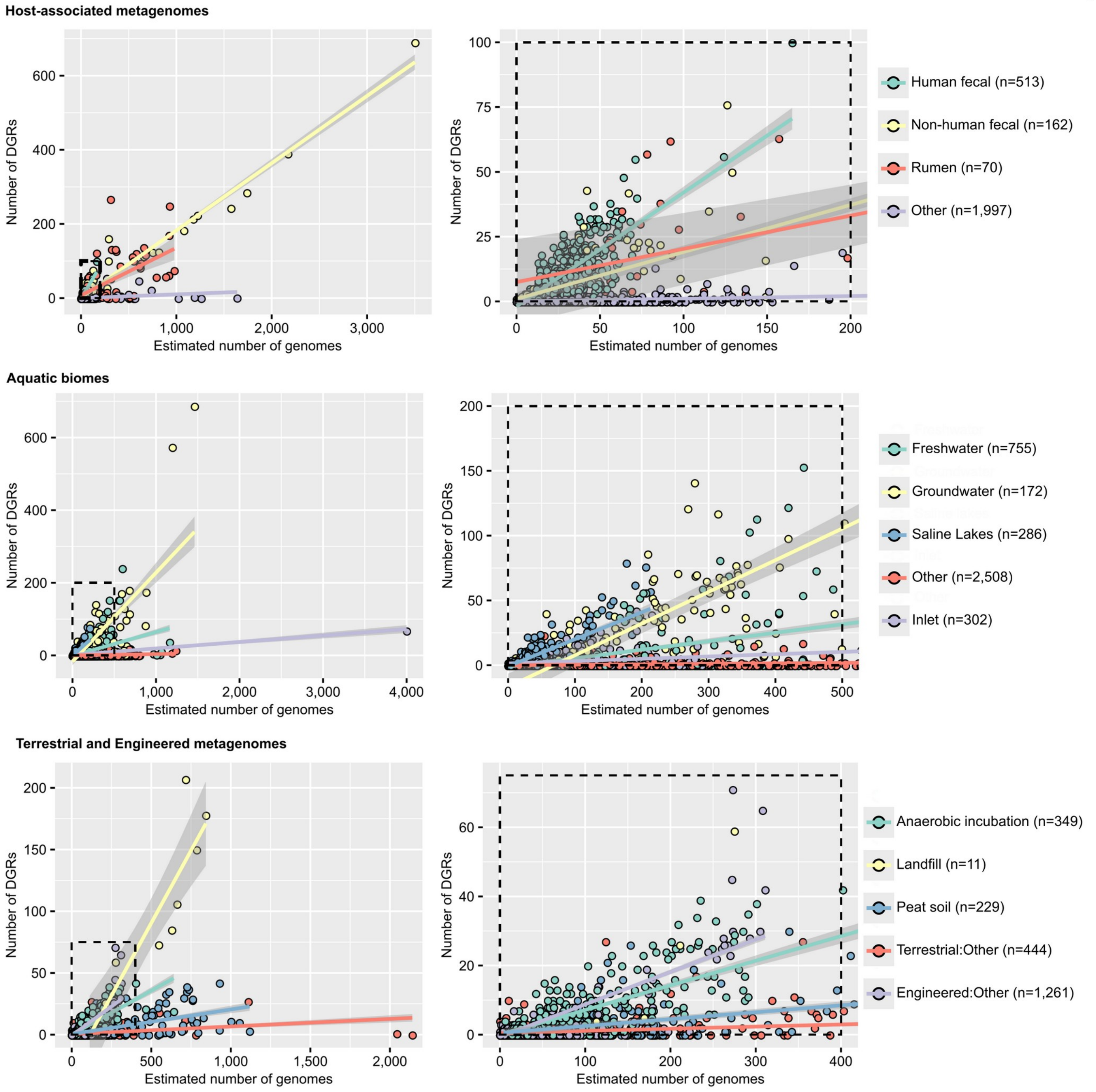

Supplementary Figure 5. **Link between estimated total number of genomes (x-axis) and number of DGRs detected (y-axis) for metagenomes across different biomes.** For each biome, a linear regression line is indicated in color, with the 95% confidence interval outlined in gray. Zoomed plots are displayed on the right panel, and the zoomed-in region is highlighted with a dashed black square on the full plot on the left panel.

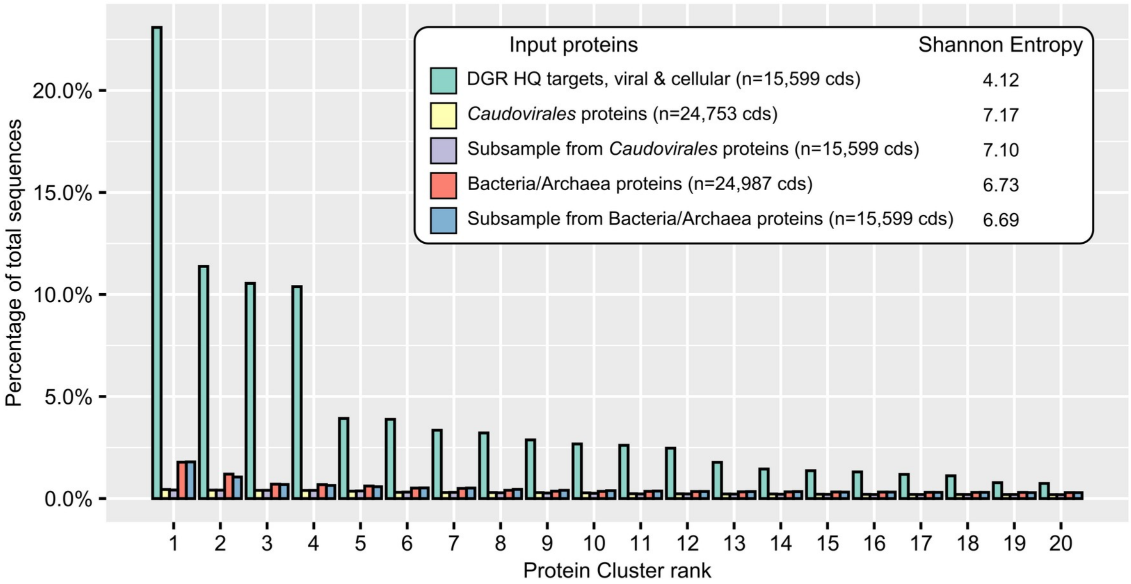

Supplementary Figure 6. **Comparison of *de novo* protein clustering for DGR targets and random microbial and/or viral sequences.** The bar chart displays the number of proteins included in each of the 20 largest PCs for 5 clustering: high-quality DGR targets, random selection of *Caudovirales* proteins (full dataset or subsampled to the same number as the HQ DGR targets), random selection of Bacteria/Archaea proteins (full dataset or subsampled to the same number as the HQ DGR targets). For each clustering, the Shannon's Entropy calculated from the entire set of clusters is also indicated in the legend box.

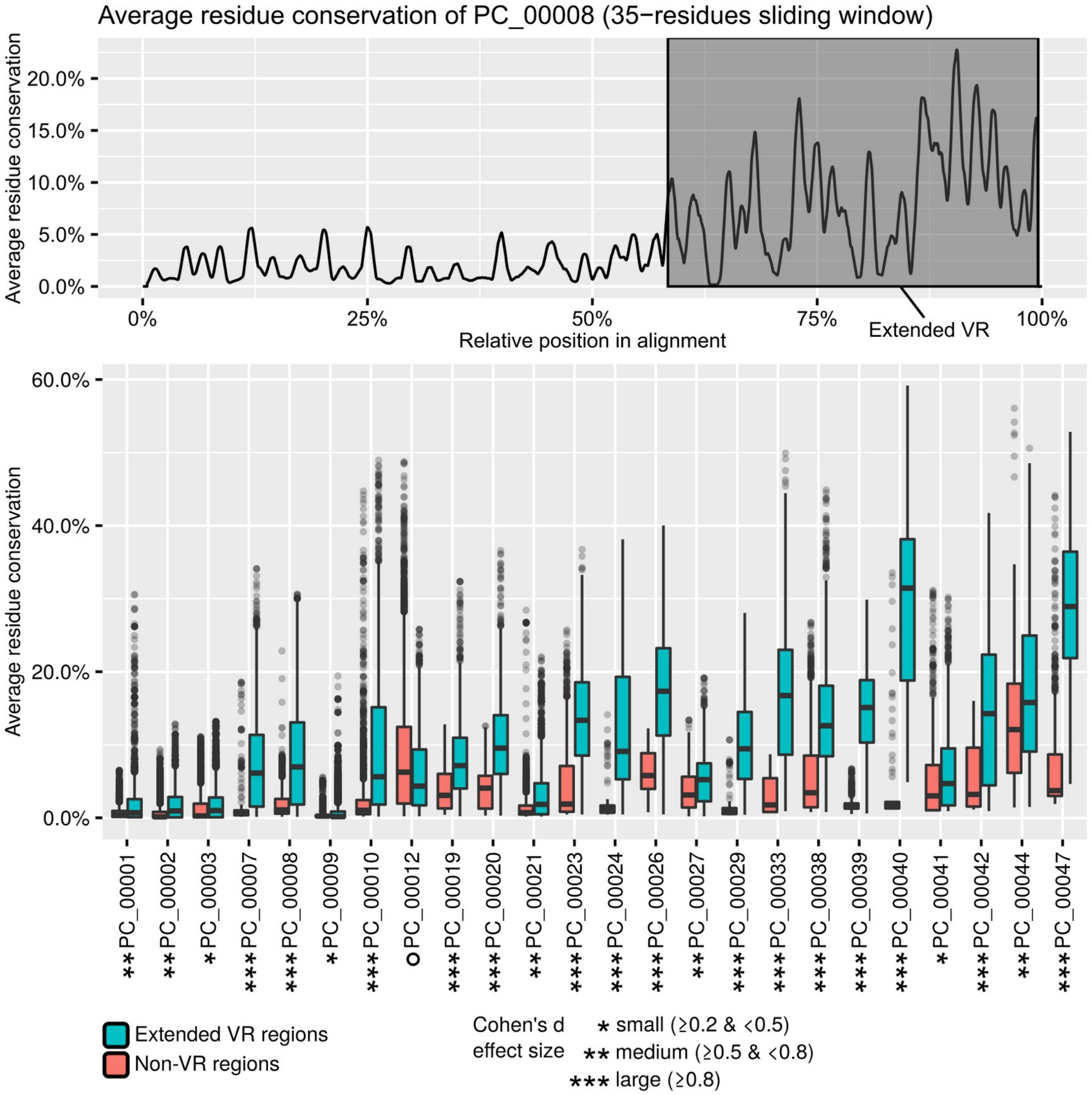

Supplementary Figure 7. **Average residue conservation in predicted targets.** A. Example of average residue conservation in 35-residues windows along the multiple alignment of PC_00008. An "extended" VR region (200 residues upstream and 20 residues downstream of the average predicted VR region) is highlighted in grey, which corresponds to the variable residues and the surrounding conserved domain. B. Distribution of residue conservation in "extended VR" and non-VR regions for the 24 largest target clusters. All distribution were significantly different (two-sided Kolmogorov–Smirnov test *p*-value <2E-16). The magnitude of the difference between VR and non-VR region is indicated through Cohen's d effect size (star symbols on the x-axis). All target PCs showed a higher average conservation in VR compared to non-VR regions except for PC_00012, which is highlighted with a black circle. The boxplot lower and upper hinges correspond to the first and third quartiles, respectively, and the whiskers extend no further than ±1.5 times the interquartile range. The numbers of observations used in the K-S tests for each PC were as follow: PC_00001: 19,082; PC_00002: 18,550; PC_00003: 11,356; PC_00007: 1,288; PC_00008: 3,467; PC_00009: 13,570; PC_00010: 3,722; PC_00012: 11,000; PC_00019: 1,911; PC_00020: 1,130; PC_00021: 8,714; PC_00023: 2,150; PC_00024: 766, PC_00026: 731, PC_00027: 3,318; PC_00029: 968; PC_00033: 748; PC_00038: 1,831; PC_00039: 1,096; PC_00040: 421; PC_00041: 3,507; PC_00042: 980; PC_00044: 1,878; and PC_00047: 1,518.

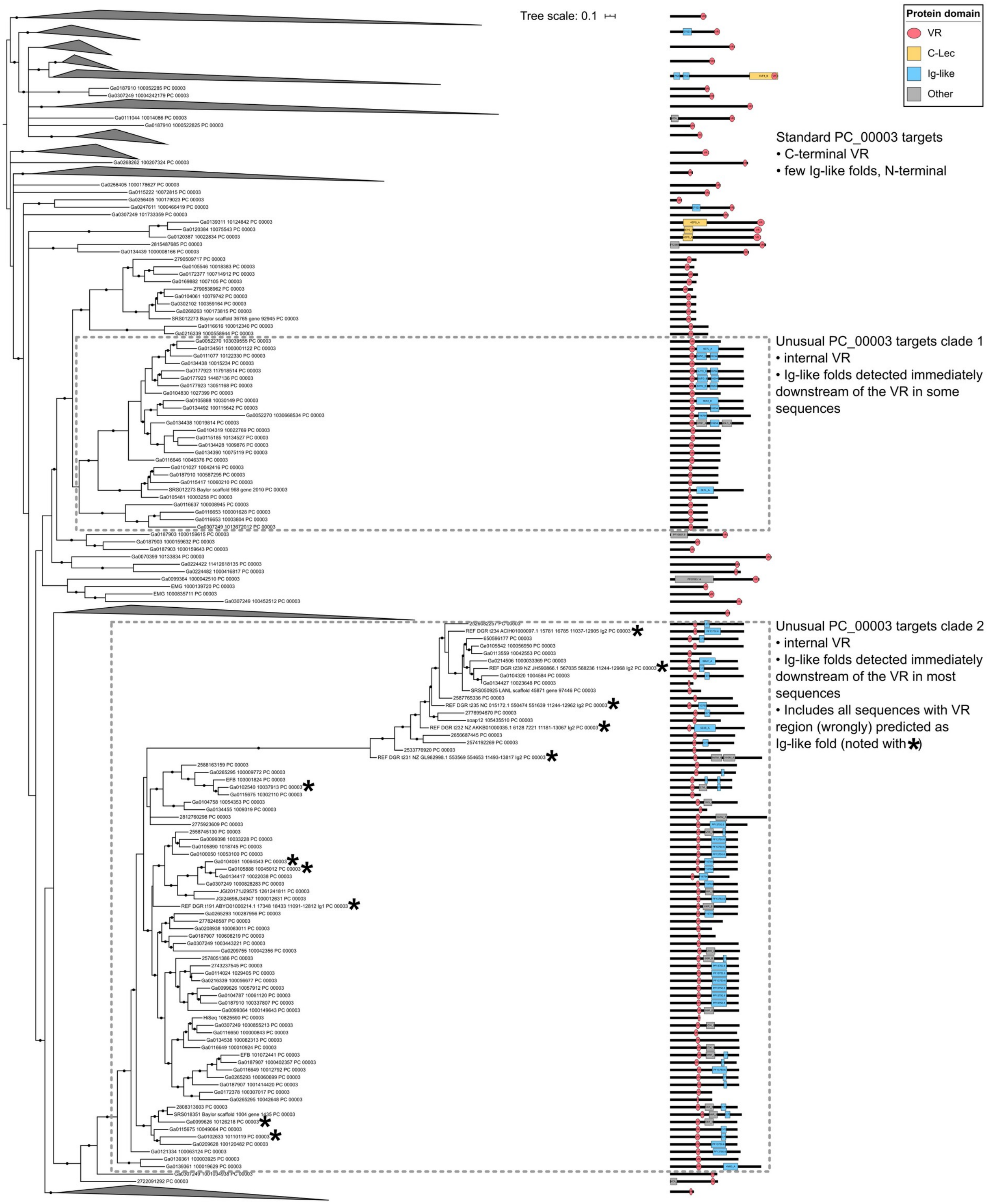

Supplementary Figure 8. **Phylogeny and domain organization of target sequences clustered in PC_00003.** Nodes with support <50 were collapsed, and nodes with support ≥80 are indicated with a black circle. For each sequence or clade, a schematic of the domain organization is indicated to the right of the tree, with a black line proportional to the sequence length, VR domains indicated with a red circle, and other domains indicated with colored rectangles. Monophyletic clades with a consistent domain organization were collapsed. The two clades of sequences displaying an internal VR region typically followed by one or several Ig-like folds in C-terminal are highlighted with a black dashed square. Reference sequences identified in the “Ig1” and “Ig2” domains are noted with a star symbol next to the sequence name.

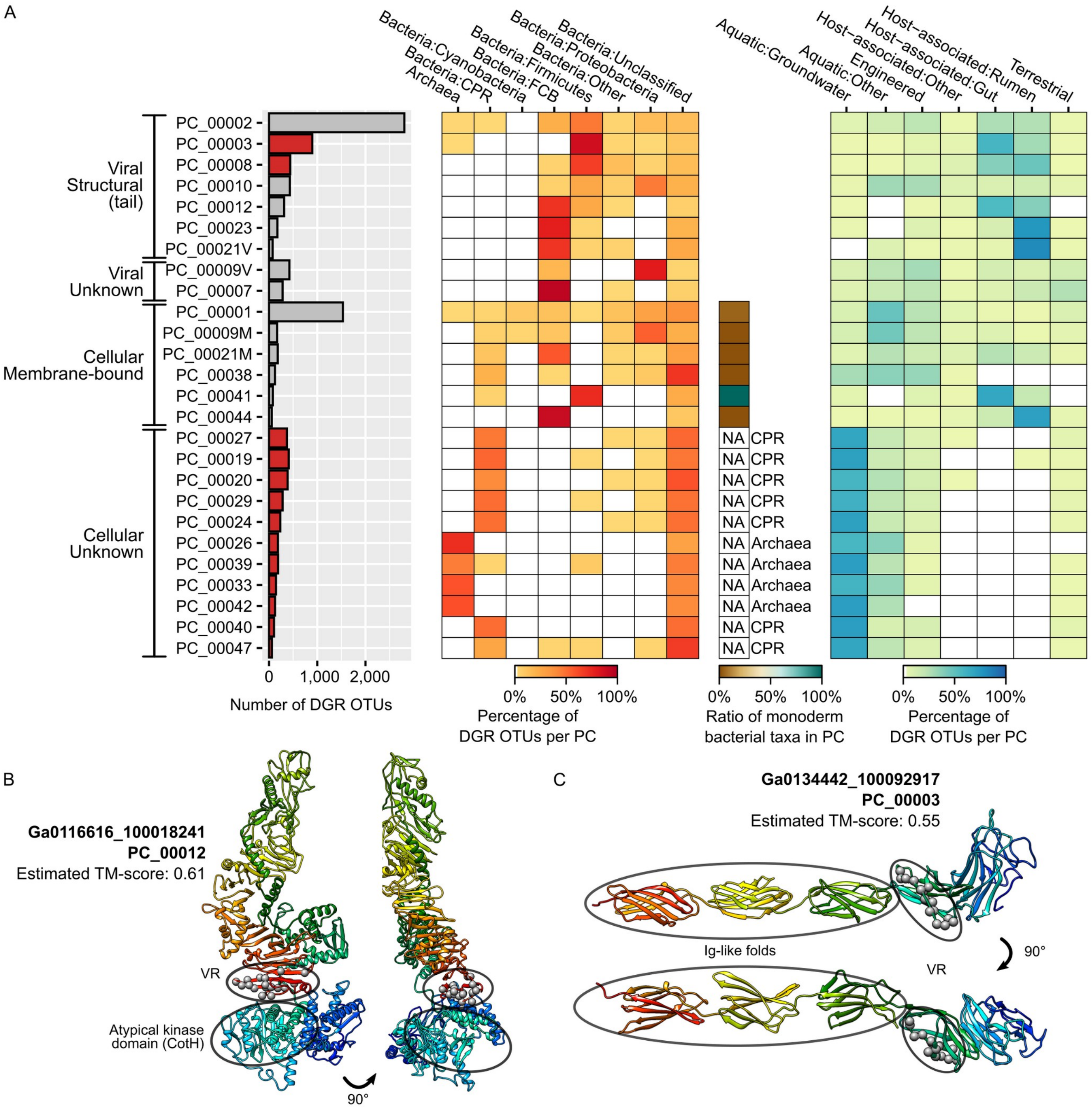

Supplementary Figure 9. **Taxonomic and biome distribution of DGR OTUs associated with the 24 largest target PCs, and predicted structure for atypical viral target.** A. PCs are ordered according to the 4 main categories of targets, as on Fig. 2. Target PCs for which the VR region was not identified as a putative C-Lec fold are highlighted in red. The proportion of DGR OTUs associated with specific taxa (left) or biomes (right) was calculated independently for each target PC. White cells in the heatmap correspond to an absence of DGR for the corresponding taxa/biome and target PC combination. For cellular-associated bacteria, the ratio of monoderm taxa to all monoderm and diderm taxa is indicated when available, based on ref. [24]. Taxa for which this membrane-based classification does not apply and/or is not available, e.g. archaea or CPR bacteria, were excluded from the analysis.

For several target PCs, this meant that almost no taxa encoding this target could be classified as "diderm bacteria" or "monoderm bacteria", and these are indicated with "NA" (<2 DGRs in taxon classified as diderm or monoderm). These target PCs were either associated with members of the CPR group or with archaea (indicated next to the "NA"). CPR: Candidate Phyla Radiation. FCB: Flavobacteria, Fibrobacteres, Chlorobi, Bacteroides. B. Predicted structure of a viral-encoded target sequence from PC_00012 displaying similarity to a eukaryotic-like kinase domain (CotH). The structure is colored with a blue-red rainbow gradient from the N- to C-terminal end and predicted variable residues in the VR (i.e., corresponding to TR adenines) are highlighted with grey spheres. Because of the large size of the protein (2,284 aa), structure prediction was run on a subset of the sequence from position 786 to 2,284, i.e., without the N-terminal region. The model quality was assessed based on the TM-score estimated by I-TASSER (a TM-score >0.5 indicates a model with a likely correct topology). C. Predicted structure of a viral-encoded target sequence from PC_00003 displaying similarity to Ig-like domains. The structure is colored with a blue-red rainbow gradient from the N- to C-terminal end and predicted variable residues in the VR (i.e., corresponding to TR adenines) are highlighted with grey spheres. The model quality was assessed based on the TM-score estimated by I-TASSER.

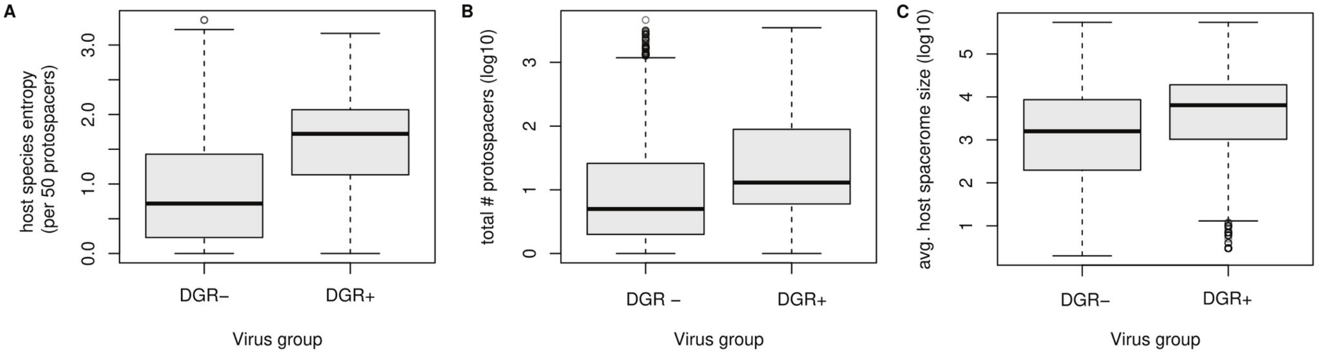

Supplementary Figure 10. **Potential indications of DGR-mediated host range expansion.** A. Species-level Shannon's Entropy of connected hosts based on a random subset 50 matched protospacers per virus. Only viral genomes matching at least 50 CRISPR spacers were considered (DGR+, n=822; DGR-, n=1,182). B. Total number of protospacers matching each type of virus with at least 1 protospacer (DGR+, n=2,566; DGR-, n=7,704). C. Number of spacer per array for connected host. For each host species, we calculated the size of the CRISPR spacer pool. For each virus, we then calculated the average of this value across all host connections, which is shown in the boxplot. DGR- viruses tend to be connected to hosts with fewer CRISPR spacers. A large pool of CRISPR spacers per species could give the appearance of narrow how range by artificially inflating the count of connections to a single species. Here instead the group with the narrower host diversity (DGR-) was associated with fewer spacers per species, suggesting this specific bias is not responsible for the lower host diversity observed for DGR- here and in Fig. 2B. For all panels, the boxplot lower and upper hinges correspond to the first and third quartiles, respectively, and the whiskers extend no further than ±1.5 times the interquartile range.

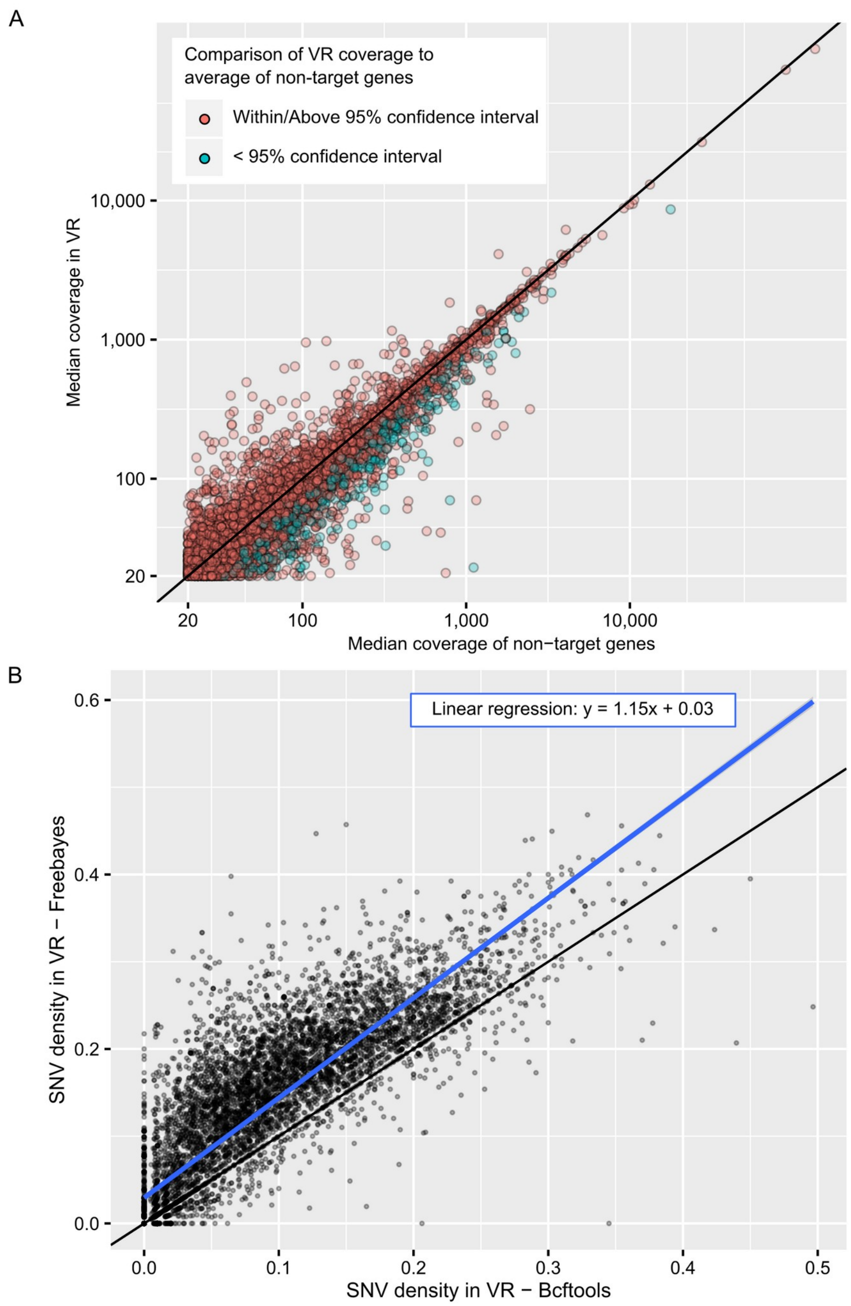

Supplementary Figure 11. **Read mapping and SNV calling on VR regions.** A. Comparison of coverage between VR regions and non-target genes for individual TR-VR pairs. Only cases with coverage ≥20x are displayed, and both x- and y-axis are displayed as log10 scale. The 1-to-1 line is indicated in black. A lower bound for a 95% confidence interval was calculated from the average coverage of non-target genes from the same contig minus 2 standard deviations. If the VR coverage was below this cutoff, it was considered as significantly lower than expected, the TR-VR was colored in blue in this plot, and flagged as "low coverage" if no SNVs were detected in Fig. 3A. B. Comparison of SNV density for individual VR regions obtained from Mpileup (x-axis) vs Freebayes (y-axis). A linear regression curve is plotted in blue, and the associated equation is indicated on the plot (p-value <2e-16).

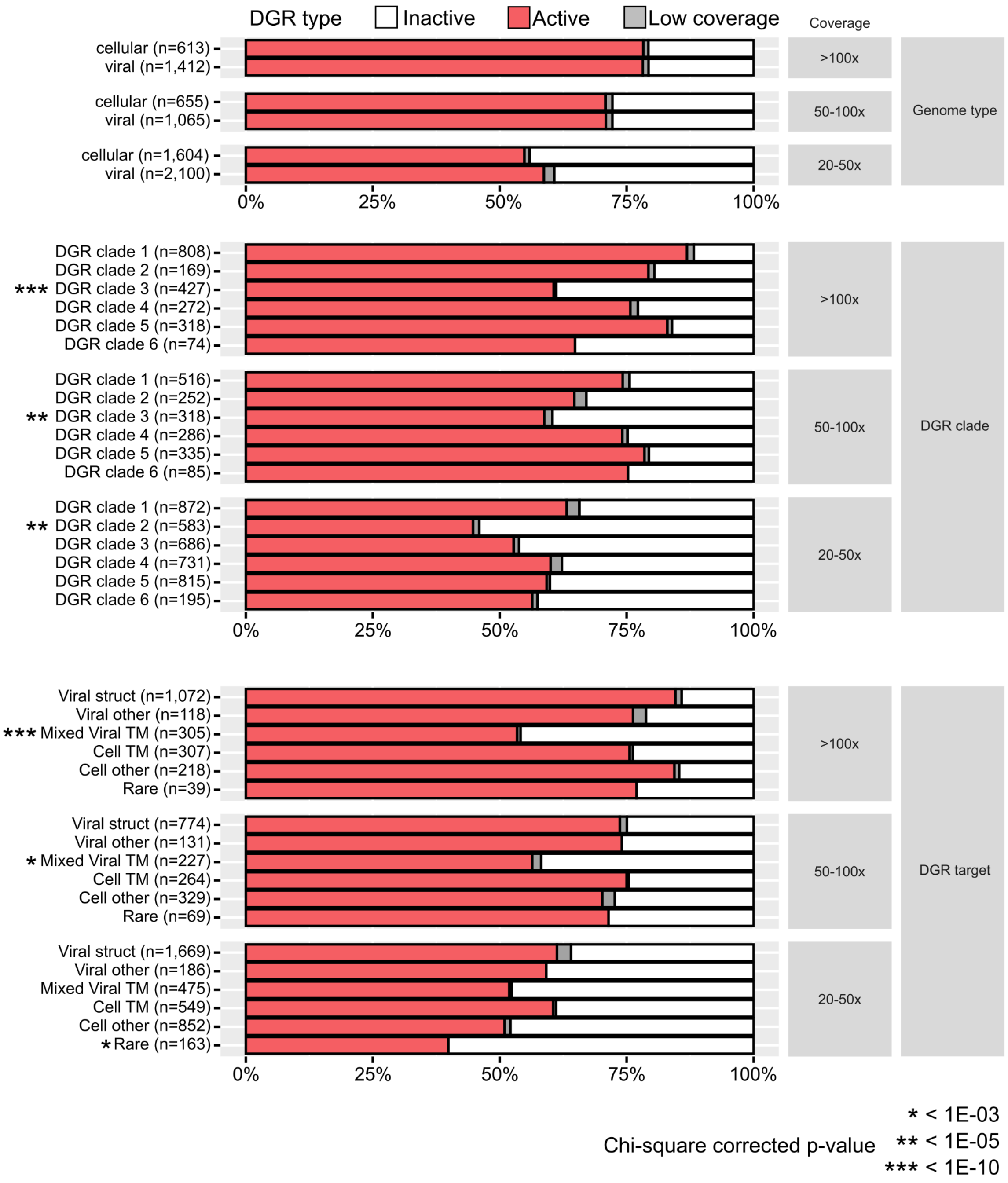

Supplementary Figure 12. **Distribution of active-vs-inactive DGRs across genome type, clade, and targets, for different ranges of coverage.** Groups (i.e., DGRs of the same genome type, DGR clade, or target) with a significantly lower proportion of active sequences compared to the average of the corresponding coverage category (Chi-squared test of independence) are highlighted with star symbols (Bonferroni-corrected *p*-values: *<1E-03, **<1E-05, ***<1E-10).

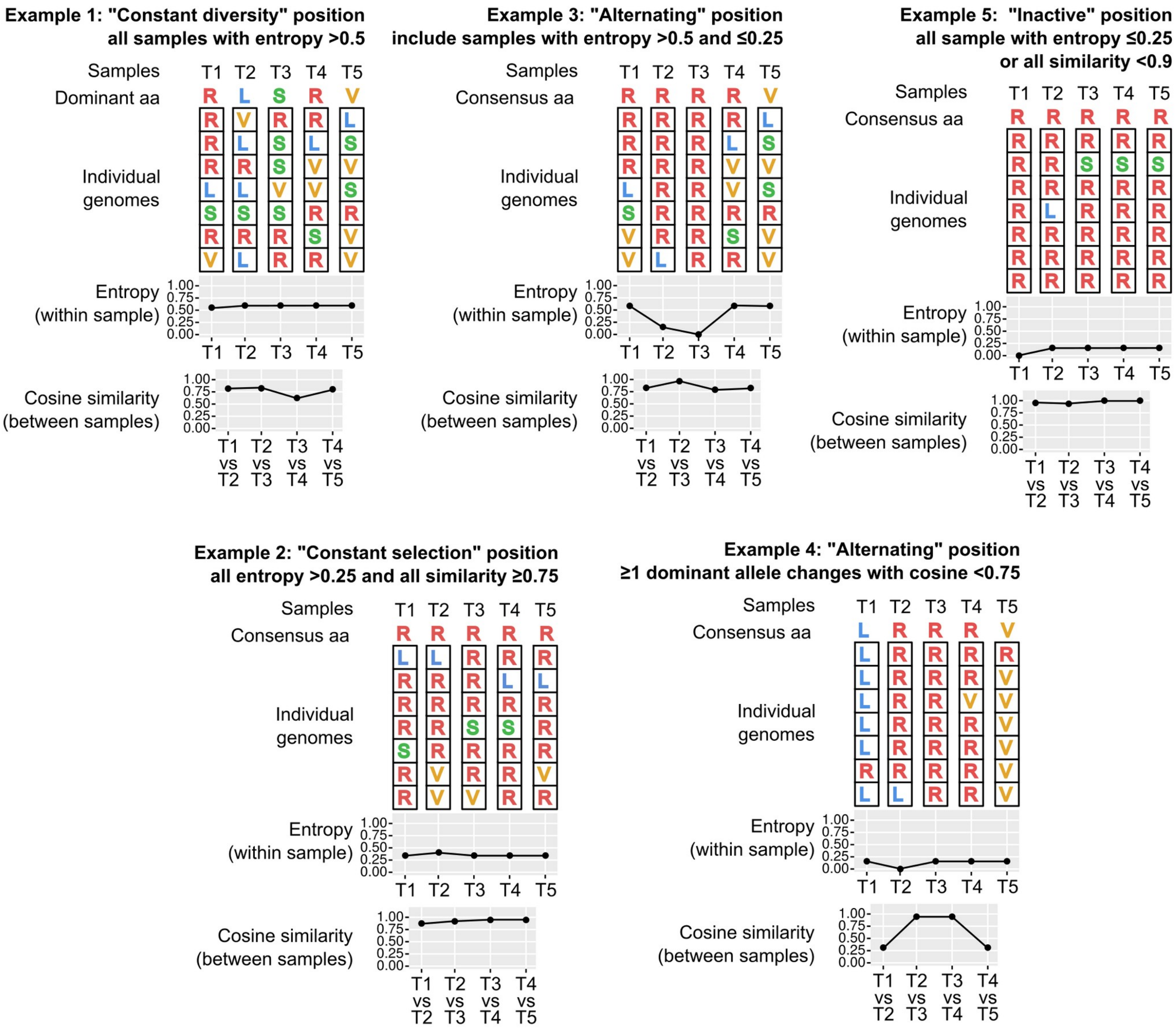

Supplementary Figure 13. **Schematic of the different categories of positions defined based on population diversity across time series.** Each example represents an individual position observed across 5 samples. The population diversity in each sample is represented as a heatmap, and the two metrics used to define the DGR activity categories are plotted underneath, either for each sample for the entropy, or between pairs of consecutive samples for the cosine similarity.

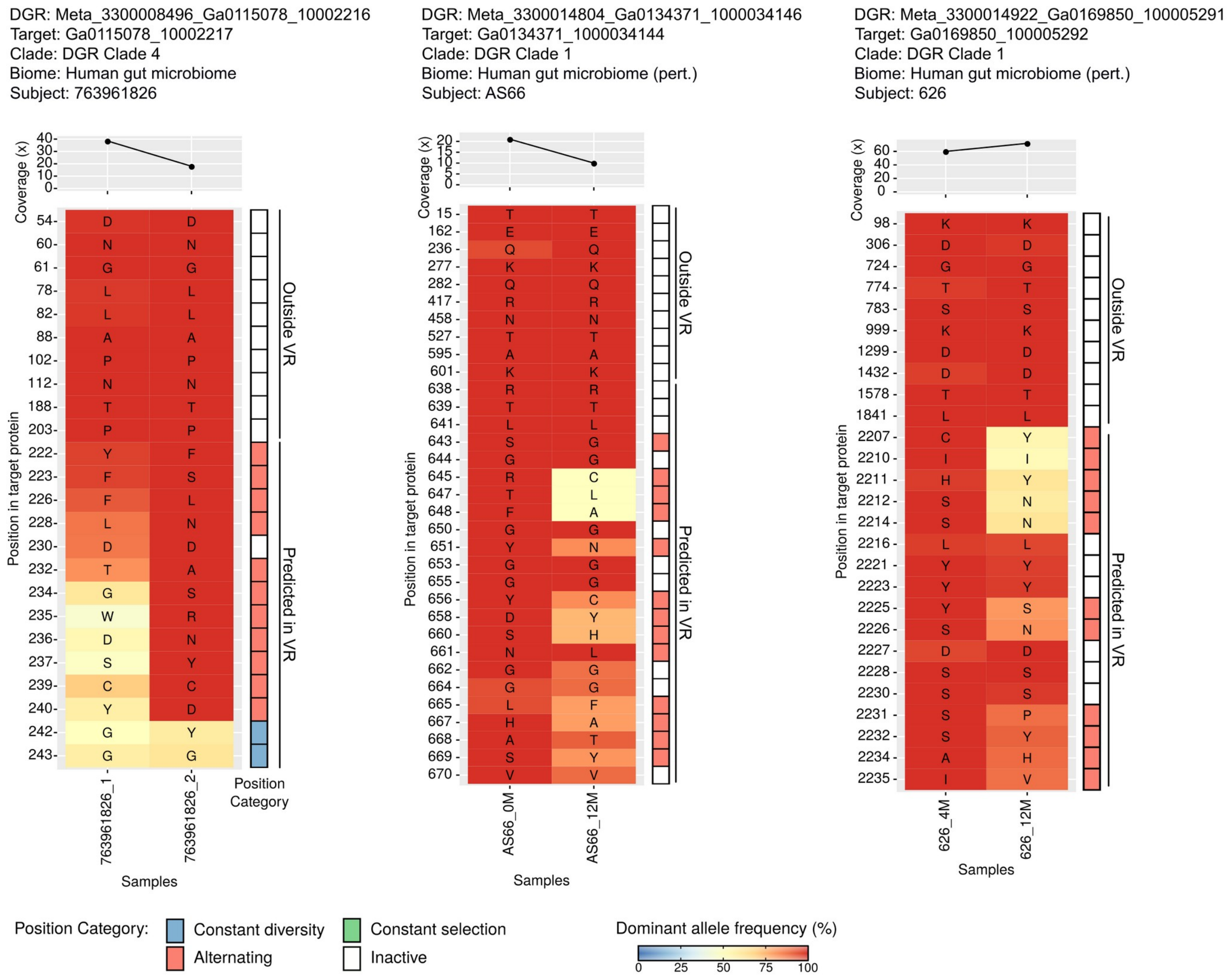

Supplementary Figure 14. **Examples of DGR target positions with changes in dominant amino acid between samples and low diversity within sample ("Alternating" pattern in Fig. 3D).** For each position (y-axis), the corresponding amino acid is indicated in the main heatmap with its frequency within the population indicated in color for each sample (x-axis). The right panel indicates the category of the position based on within-sample entropy, between-samples cosine distances, and number of amino acid changes in the time series (see Supplementary Note 12), colored as in Fig. 3D. The top panel indicates the median coverage of all positions in each sample. For reference purposes, 10 random positions from the same protein but outside of the predicted VR are included in the heatmap.

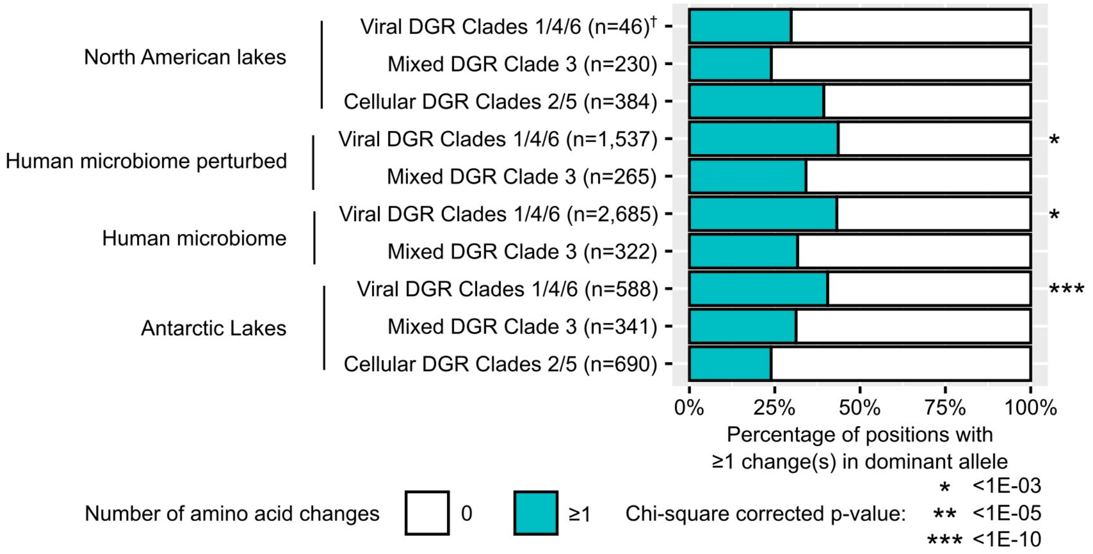

Supplementary Figure 15. **Percentage of positions with ≥1 change(s) in dominant allele among positions considered as “Constant diversity” or “Alternating” for different types of DGR across major biomes.** For each biome, the percentage in “Viral structural – Clades 1/4/6” DGRs was compared to the percentage in other DGR categories combined using a Chi-square test of independence. Groups with a significantly higher proportion of positions with ≥1 change(s) are highlighted with star symbols (Bonferroni-corrected *p*-values: *<1E-03, **<1E-05, ***<1E-10). † Counts for DGRs associated with viral structural proteins in temperate lakes are based on only 5 DGRs, while all other environments had >10 DGRs associated with viral structural proteins.

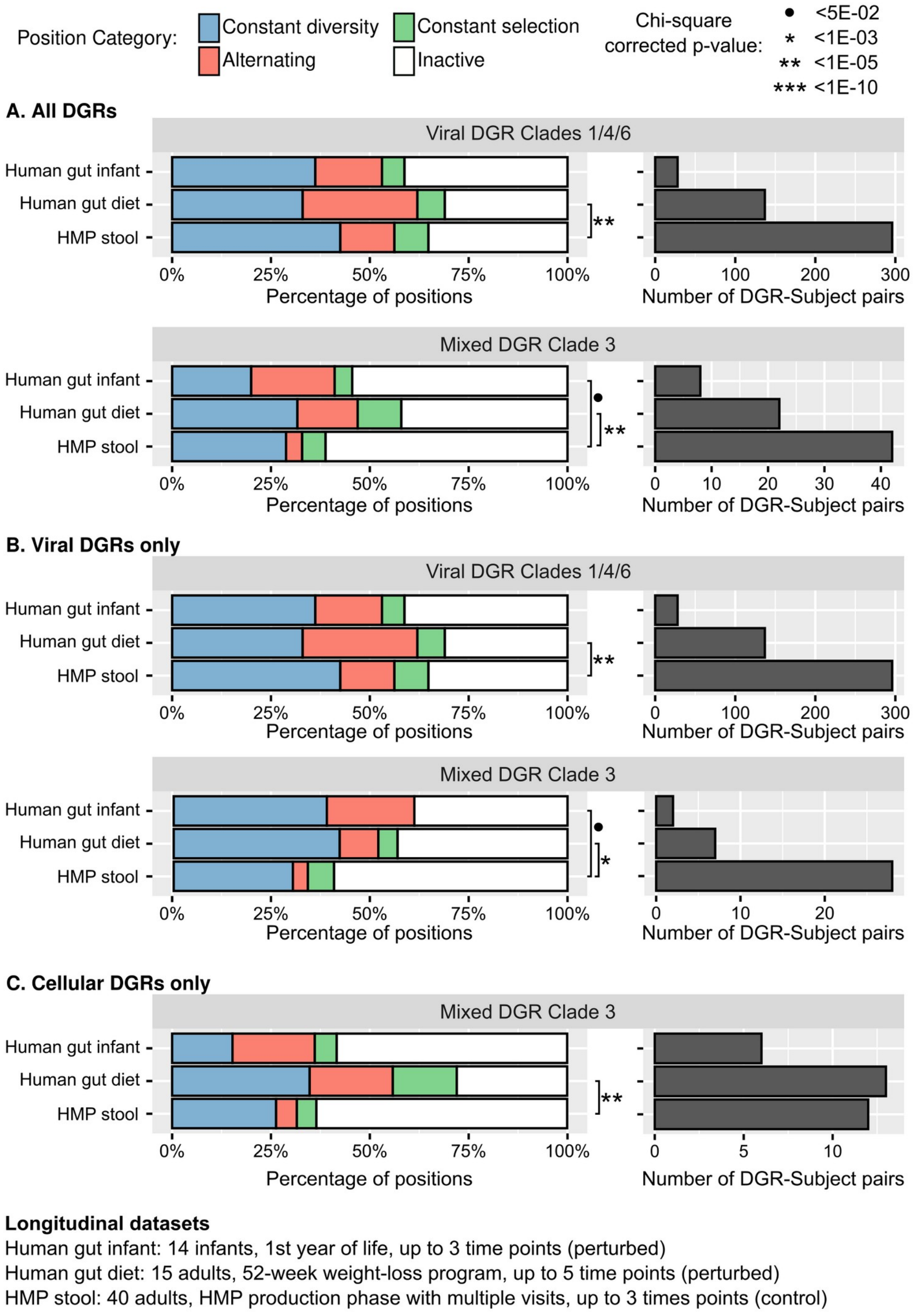

Supplementary Figure 16. **Comparison of DGR activity between infant, perturbed, and non-perturbed human microbiome samples.** Left panels display the distribution of activity categories for VR positions between infant, perturbed, and non-perturbed human gut microbiome DGRs. The conditions under which each dataset was collected are indicated at the bottom of the figure. The right panel bar graph indicates the number of observations (i.e., total number of DGRs covered in at least 2 time points across all subjects) for each dataset. Panel A includes all relevant DGRs, while panel B and C include only viral- or cellular-encoded DGRs, respectively. Statistically significant comparisons (Chi-square of independence) are highlighted with star symbols (Bonferroni-corrected *p*-values: <5E-02 *<1E-03, **<1E-05, ***<1E-10).

Supplementary Table

**Cellular DGRs (GEM dataset) – rpoB tree**					
		mean	95% CI	p-value	Note
Phylogenetic signal	a parameter (=-log(α))	-2.359	-1.969;1.115	< 0.002	bootstrap mean: -1.045 so possible downward bias
Biome signal	Aquatic:Groundwater	-0.222	-0.597;0.344	0.502	
	Aquatic:Inlet	-0.269	-0.957;0.284	0.541	
	**Aquatic:Other**	**-1.591**	**-2.219;-0.519**	**2.33E-05**	
	Aquatic:Saline-lakes	-0.161	-0.791;0.359	0.704	
	Engineered:Anaerobic-incubation	-0.434	-0.678;0.084	0.123	
	Engineered:Landfill	-0.716	-1.376;0.152	0.231	
	Engineered:Other	-1.745	-2.472;-0.224	0.005	
	Host-associated:Human-fecal	0.410	0.005;0.937	0.201	
	Host-associated:NonHuman-fecal	-0.257	-0.665;0.397	0.498	
	Host-associated:Other	-0.472	-0.776;0.031	0.121	
	Terrestrial:Other	-1.101	-1.715;-0.121	0.008	
	Terrestrial:Soil-peat	-0.306	-0.963;0.666	0.657	
**Viral DGRs (IMG/VR dataset) – TerL tree**					
		mean	95% CI	p-value	Note
Phylogenetic signal	a parameter (=-log(α))	-0.788	-0.629;0.183	< 0.002	bootstrap mean: -0.207 so possible downward bias
Biome signal	Aquatic:Groundwater	0.656	0.388;1.139	0.046	
	Aquatic:Inlet	-0.341	-0.682;0.138	0.516	
	Aquatic:Other	0.212	-0.071;0.682	0.498	
	Aquatic:Saline-lakes	0.938	0.581;1.341	0.006	
	Engineered:Anaerobic-incubation	0.929	0.671;1.391	0.003	
	**Engineered:Landfill**	**1.193**	**0.939;1.529**	**1.77E-04**	
	Engineered:Other	0.566	0.275;1.057	0.076	
	**Host-associated:Human-fecal**	**1.614**	**1.492;1.936**	**2.24E-08**	
	**Host-associated:NonHuman-fecal**	**1.791**	**1.592;2.055**	**2.11E-10**	
	**Host-associated:Other**	**1.313**	**1.244;1.546**	**1.08E-06**	
	Terrestrial:Other	0.177	-0.113;0.722	0.669	
	Terrestrial:Soil-peat	0.627	0.33;1.181	0.103	

Supplementary Table 1. **Results of phylogenetic logistic regression analysis on DGR trees.** For this analysis, DGRs were first connected to metagenome-assembled genomes (both microbial and viral). Trees were then constructed using genome marker genes: RNA polymerase B for microbes (RpoB), and Terminase Large subunit (TerL) for viruses. Trees included 174 and 883 DGR-encoding genomes for RpoB and TerL, respectively, along sequences from non-DGR-encoding related genomes (see Methods). A binary phylogenetic regression was run for each tree using the presence of a DGR as the

dependent variable, and the ecosystem category as independent variable. For each tree, the table includes the strength of the phylogenetic signal (a parameter) and the correlation coefficient observed for each ecosystem category, along with 95% confidence intervals and *p*-values based on 5,000 boostraps. Ecosystem categories considered here to be significantly correlated with DGR distribution are highlighted in bold.

REFERENCES

1. Schulz, F. *et al.* Giant virus diversity and host interactions through global metagenomics. *Nature* **578**, (2020).
2. Roux, S. *et al.* Cryptic inoviruses revealed as pervasive in bacteria and archaea across Earth's biomes. *Nat. Microbiol.* **4**, 1895–1906 (2019).
3. Wu, L. *et al.* Diversity-generating retroelements : natural variation , classification and evolution inferred from a large-scale genomic survey. *Nucleic Acids Res.* **46**, 11–24 (2018).
4. Liu, M. *et al.* Reverse transcriptase-mediated tropism switching in Bordetella bacteriophage. *Science (80-.).* **295**, 2091–4 (2002).
5. Le Coq, J. & Ghosh, P. Conservation of the C-type lectin fold for massive sequence variation in a Treponema diversity-generating retroelement. *Proc. Natl. Acad. Sci.* **108**, 14649–14653 (2011).
6. Paul, B. G. *et al.* Targeted diversity generation by intraterrestrial archaea and archaeal viruses. *Nat. Commun.* **6**, 6585 (2015).
7. Arambula, D. *et al.* Surface display of a massively variable lipoprotein by a Legionella diversity-generating retroelement. *Proc. Natl. Acad. Sci.* **110**, 8212–8217 (2013).
8. Paul, B. G. *et al.* Retroelement-guided protein diversification abounds in vast lineages of Bacteria and Archaea. *Nat. Microbiol.* **2**, 17045 (2017).
9. Handa, S. *et al.* Template-assisted synthesis of adenine-mutagenized cDNA by a retroelement protein complex. *Nucleic Acids Res.* **46**, 9711–9725 (2018).
10. Ives, A. R. & Garland, T. Phylogenetic logistic regression for binary dependent variables. *Syst. Biol.* **59**, 9–26 (2010).
11. Canchaya, C., Fournous, G., Chibani-Chennoufi, S., Dillmann, M. L. & Brüssow, H. Phage as agents of lateral gene transfer. *Curr. Opin. Microbiol.* **6**, 417–424 (2003).
12. Roux, S., Hallam, S. J., Woyke, T. & Sullivan, M. B. Viral dark matter and virus-host interactions resolved from publicly available microbial genomes. *Elife* **4**, e08490 (2015).
13. Iranzo, J., Krupovic, M. & Koonin, E. V. The double-stranded DNA virosphere as a modular hierarchical network of gene sharing. *MBio* **7**, e00978-16 (2016).
14. O'Leary, N. A. *et al.* Reference sequence (RefSeq) database at NCBI: Current status, taxonomic expansion, and functional annotation. *Nucleic Acids Res.* **44**, D733–D745 (2016).
15. Fraser, J. S., Yu, Z., Maxwell, K. L. & Davidson, A. R. Ig-Like Domains on Bacteriophages: A Tale of Promiscuity and Deceit. *J. Mol. Biol.* **359**, 496–507 (2006).

16. Nguyen, K. B. *et al.* Phosphorylation of spore coat proteins by a family of atypical protein kinases. *Proc. Natl. Acad. Sci. U. S. A.* **113**, E3482–E3491 (2016).

17. Park, B. C., Reese, M., Vincent, S. & Tagliabracci, V. S. Thinking outside of the cell: Secreted protein kinases in bacteria, parasites, and mammals. *IUBMB Life* **71**, 749–759 (2019).

18. Abid, D. & Zhang, L. DeepCapTail: A Deep Learning Framework to Predict Capsid and Tail Proteins of Phage Genomes. *bioRxiv* 1–14 (2018) doi:10.1101/477885.

19. Cantu, V. A. *et al.* PhANNs, a fast and accurate tool and web server to classify phage structural proteins. *PLoS Comput. Biol.* **16**, 1–18 (2020).

20. Lauro, F. M. *et al.* The genomic basis of trophic strategy in marine bacteria. *Proc. Natl. Acad. Sci. U. S. A.* **106**, 15527–15533 (2009).

21. Roux, S. *et al.* Optimizing de novo genome assembly from PCR-amplified metagenomes. *PeerJ* **2019**, 1–18 (2019).

22. Schloissnig, S. *et al.* Genomic variation landscape of the human gut microbiome. *Nature* **493**, 45–50 (2013).

23. Delmont, T. O. *et al.* Single-amino acid variants reveal evolutionary processes that shape the biogeography of a global SAR11 subclade. *Elife* **8**, 1–26 (2019).

24. Gupta, R. S. Origin of diderm (Gram-negative) bacteria: Antibiotic selection pressure rather than endosymbiosis likely led to the evolution of bacterial cells with two membranes. *Antonie van Leeuwenhoek, Int. J. Gen. Mol. Microbiol.* **100**, 171–182 (2011).
