# Supplementary material for: Ecology and molecular targets of hypermutation in the global microbiome
Source: Nat Commun. 2021 May 24;12:3076. doi: 10.1038/s41467-021-23402-7 (PMC8144416; doi:10.1038/s41467-021-23402-7)
Supplement: Supplementary file 3 — Description of Additional Supplementary Files [file 41467_2021_23402_MOESM3_ESM.docx]

**Ecology and molecular targets of hypermutation in the global microbiome.**

Description of Additional Supplementary Files

File Name: Supplementary Data 1

Description: List of metagenomes mined for DGRs, including dataset and sample identifiers, ecological category, sample type, and publication based on information from IMG and Gold databases.

File Name: Supplementary Data 2

Description: List of DGRs detected in public genomes and metagenomes, including structural, taxonomy, and ecosystem annotation.

File Name: Supplementary Data 3

Description: List of DGR OTUs with annotation, including genome type, taxonomy, biome, and target.

File Name: Supplementary Data 4

Description: List of DGR clusters with annotation, including genome type, taxonomy, biome, and target.

File Name: Supplementary Data 5

Description: List of predicted DGR targets with structural annotation and associated benchmarks.

File Name: Supplementary Data 6

Description: List of longitudinal datasets used in the time-series analyses of DGR-induced sequence variation.

File Name: Supplementary Data 7

Description: Fasta file of amino acid sequences for all RTs used in the analysis (DGR and non DGR)

File Name: Supplementary Data 8

Description: Fasta file of amino acid sequences for all predicted DGR targets.

File Name: Supplementary Data 9

Description: Fasta file of nucleotide sequences for TR/VR pairs.
