# Supplementary material for: Ecology and molecular targets of hypermutation in the global microbiome
Source: Nat Commun. 2021 May 24;12:3076. doi: 10.1038/s41467-021-23402-7 (PMC8144416; doi:10.1038/s41467-021-23402-7)
Supplement: Supplementary file 13 — Reporting Summary [file 41467_2021_23402_MOESM13_ESM.pdf]

nature research

Corresponding author(s): Simon Roux
Emiley Eloe-Fadrosh

Last updated by author(s): Mar 25, 2021

Reporting Summary

Nature Research wishes to improve the reproducibility of the work that we publish. This form provides structure for consistency and transparency in reporting. For further information on Nature Research policies, see our Editorial Policies and the Editorial Policy Checklist.

Statistics

For all statistical analyses, confirm that the following items are present in the figure legend, table legend, main text, or Methods section.

n/a	Confirmed	
☐	☒	The exact sample size (n) for each experimental group/condition, given as a discrete number and unit of measurement
☒	☐	A statement on whether measurements were taken from distinct samples or whether the same sample was measured repeatedly
☐	☒	The statistical test(s) used AND whether they are one- or two-sided<br>*Only common tests should be described solely by name; describe more complex techniques in the Methods section.*
☐	☒	A description of all covariates tested
☐	☒	A description of any assumptions or corrections, such as tests of normality and adjustment for multiple comparisons
☐	☒	A full description of the statistical parameters including central tendency (e.g. means) or other basic estimates (e.g. regression coefficient) AND variation (e.g. standard deviation) or associated estimates of uncertainty (e.g. confidence intervals)
☐	☒	For null hypothesis testing, the test statistic (e.g. F, t, r) with confidence intervals, effect sizes, degrees of freedom and P value noted<br>*Give P values as exact values whenever suitable.*
☒	☐	For Bayesian analysis, information on the choice of priors and Markov chain Monte Carlo settings
☒	☐	For hierarchical and complex designs, identification of the appropriate level for tests and full reporting of outcomes
☐	☒	Estimates of effect sizes (e.g. Cohen's d, Pearson's r), indicating how they were calculated

Our web collection on statistics for biologists contains articles on many of the points above.

Software and code

Policy information about availability of computer code

Data collection	No software was used to collect data for this study
Data analysis	Code availability:<br>Custom scripts used in this study are available at http://bitbucket.org/srouxjgi/dgr_scripts/. The version used in this manuscript corresponds to the "v1.0" release/tag.<br><br>Other tools used in the study:<br>- hmmsearch v3.2.1<br>- blastn and blastp v2.9.0+<br>- cd-hit v4.8.1<br>- FastTree v2<br>- Muscle v3.8<br>- HMMER v3.2.1<br>- VirSorter v1.0.5<br>- vContact2 v0.9.10<br>- mcl v14-137<br>- Anvi'o v6.1<br>- R v3.6.1<br>- HHsuite v3.1.0<br>- TMHMM v2.0c<br>- SignalP v4.1<br>- DeepCapTail v3038c4d

- PhANNs v1.0.0
- I-TASSER v5.1
- metabat v0.32.4
- checkm v1
- gtdb-tk v0.3
- CRT v1.1
- PILER-CR v1.0.6
- CheckV v0.7.0
- MAFFT v7.407
- TrimAL v1.4.rev15
- IQ-Tree v1.5.5
- R package phytools v0.6-99
- R package phylolm v2.6.2
- bwa v0.7.17-r1188
- bbmap v38.73
- bcftools v1.9
- FreeBayes v1.3.1
- Anvi'o v6.1
- R package ggplot2 v3.2.1
- iToL v4
- UCSF Chimera v1.11.2
- GNU parallel v20190722
- Easyfig v2.2.3

For manuscripts utilizing custom algorithms or software that are central to the research but not yet described in published literature, software must be made available to editors and reviewers. We strongly encourage code deposition in a community repository (e.g. GitHub). See the Nature Research guidelines for submitting code & software for further information.

Data

Policy information about availability of data

All manuscripts must include a data availability statement. This statement should provide the following information, where applicable:

- Accession codes, unique identifiers, or web links for publicly available datasets
- A list of figures that have associated raw data
- A description of any restrictions on data availability

All metagenome assemblies are available through IMG (https://img.jgi.doe.gov) using accession numbers listed in Supplementary Table 1.
Other databases used in the study include:
- Pfam v31 (http://pfam.xfam.org/)
- NCBI GenBank (sequences downloaded on January 23 2019) - https://www.ncbi.nlm.nih.gov/
- Pdb79 v 190918, Pfam v32, and SCOPe70 v1.75, all provided as part of the HH-suite package (http://wwwuser.gwdg.de/~compbiol/data/hhsuite/databases/hhsuite_dbs/)
- Gold (i.e. Genome Online Database - gold.jgi.doe.gov), accessed on April 19, 2019

Field-specific reporting

Please select the one below that is the best fit for your research. If you are not sure, read the appropriate sections before making your selection.

☒ Life sciences ☐ Behavioural & social sciences ☐ Ecological, evolutionary & environmental sciences

For a reference copy of the document with all sections, see nature.com/documents/nr-reporting-summary-flat.pdf

Life sciences study design

All studies must disclose on these points even when the disclosure is negative.

Sample size	No sample-size calculation was performed. The dataset was gathered from a total of 1,129 genomes and 2,684 metagenomes, mining publicly available data from the IMG website. All datasets publicly available at the time were included in the study, so the sample size could not be increased. The number of DGRs obtained (> 30,000) and their diversity being ~ 15 times larger than any other study published previously, the sample size was judged sufficient for the type of analysis performed.
Data exclusions	No data was excluded
Replication	All analyses were computational, and were reproduced at least twice in silico, always with a successful replication of the results.
Randomization	None of the analyses required randomization
Blinding	None of the analyses required random grouping, so that there was no need for any blinding. Since the analysis were primarily exploratory, there was no expected trend in the distribution of the data and/or of the samples, and no test of e.g. the effect in one group vs the other. Hence there was no parameter that could be retained from the investigators, and no assumption from the part of the investigator that could

cause some issue in the interpretation of the results.

Reporting for specific materials, systems and methods

We require information from authors about some types of materials, experimental systems and methods used in many studies. Here, indicate whether each material, system or method listed is relevant to your study. If you are not sure if a list item applies to your research, read the appropriate section before selecting a response.

Materials & experimental systems

n/a	Involved in the study
☒	☐ Antibodies
☒	☐ Eukaryotic cell lines
☒	☐ Palaeontology and archaeology
☒	☐ Animals and other organisms
☒	☐ Human research participants
☒	☐ Clinical data
☒	☐ Dual use research of concern

Methods

n/a	Involved in the study
☒	☐ ChIP-seq
☒	☐ Flow cytometry
☒	☐ MRI-based neuroimaging
