# Supplementary material for: Ecology and molecular targets of hypermutation in the global microbiome
Source: Nat Commun. 2021 May 24;12:3076. doi: 10.1038/s41467-021-23402-7 (PMC8144416; doi:10.1038/s41467-021-23402-7)
Supplement: Supplementary file 2 — Peer Review File [file 41467_2021_23402_MOESM2_ESM.pdf]

Editorial Note: This manuscript has been previously reviewed at another journal that is not operating a transparent peer review scheme. This document only contains reviewer comments and rebuttal letters for versions considered at *Nature Communications.*

REVIEWERS' COMMENTS

Reviewer #1 (Remarks to the Author):

The authors have adequately addressed our concerns and the manuscript is much improved in response to comments from all three reviewers. This work will be of interest to many readers and I look forward to seeing it published.

Reviewer #2 (Remarks to the Author):

The authors replied all my comments and made all necessary edits. I don't have further comments on the paper.

Reviewer #3 (Remarks to the Author):

The authors have performed a substantial amount of new analysis, addressing all of my previous concerns. The authors have also integrated their novel analysis into the main and supplemental texts, and have refined their conclusions accordingly. I support publication of the paper as is in its current form

REVIEWERS' COMMENTS

Reviewer #1 (Remarks to the Author):

The authors have adequately addressed our concerns and the manuscript is much improved in response to comments from all three reviewers. This work will be of interest to many readers and I look forward to seeing it published.

Reviewer #2 (Remarks to the Author):

The authors replied all my comments and made all necessary edits. I don't have further comments on the paper.

Reviewer #3 (Remarks to the Author):

The authors have performed a substantial amount of new analysis, addressing all of my previous concerns. The authors have also integrated their novel analysis into the main and supplemental texts, and have refined their conclusions accordingly. I support publication of the paper as is in its current form

We thank all 3 reviewers for their comments which undoubtedly led to substantial improvements in the manuscript.
